# Supplementary material for: Efficient Synthesis of New Fluorinated β-Amino Acid Enantiomers through Lipase-Catalyzed Hydrolysis
Source: Molecules. 2020 Dec 17;25(24):5990. doi: 10.3390/molecules25245990 (PMC7766834; doi:10.3390/molecules25245990)

Supporting Information

Efficient synthesis of new fluorinated β-amino acid enantiomers through lipase-catalyzed hydrolysis

Sayeh Shahmohammadi; Ferenc Fülöp; Enikő Forró

Figure 1. ^{1}H NMR (500 MHz, D_2O, 25 °C) spectra for (±) **2a**

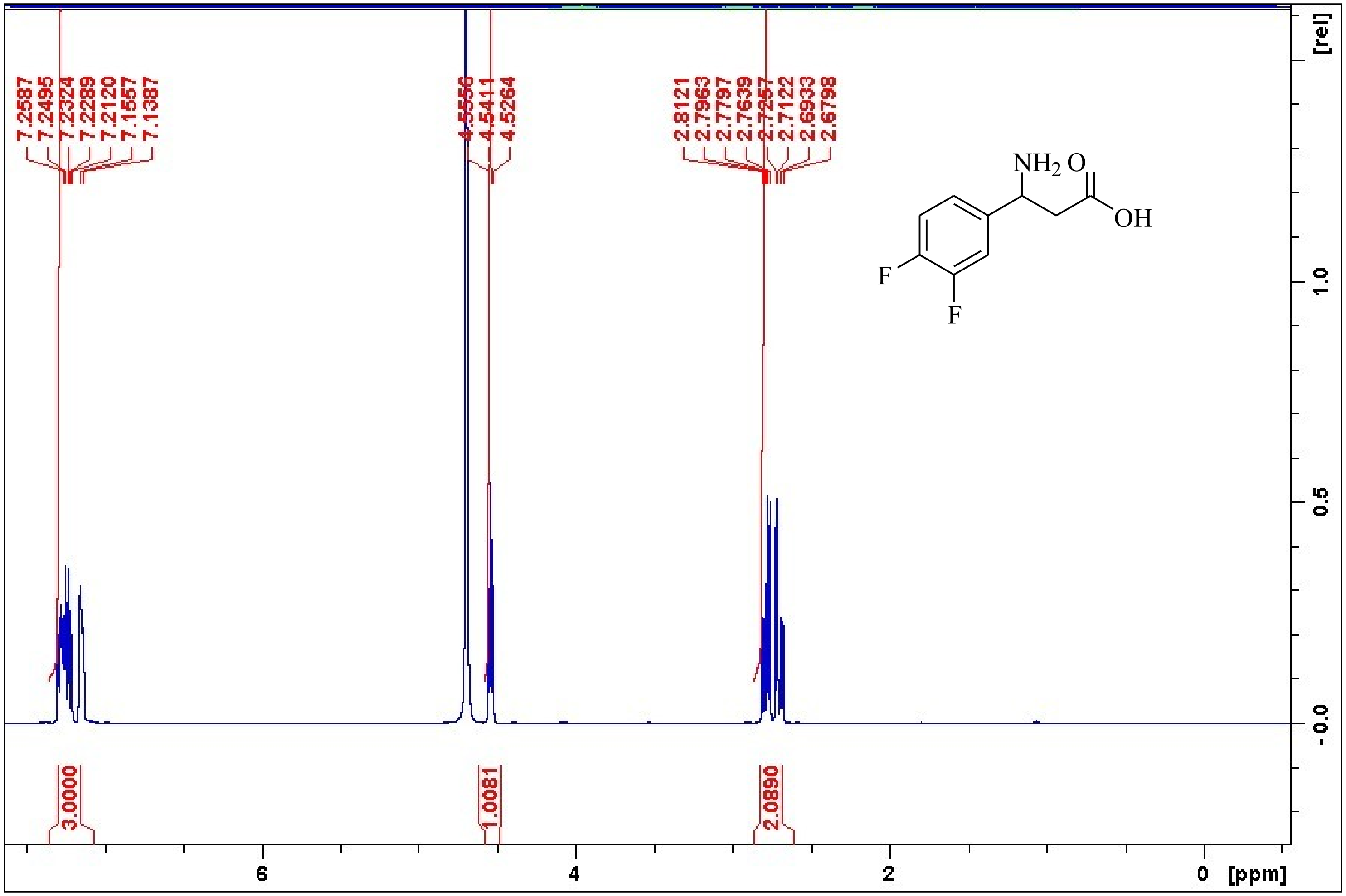

Figure 2. ^{1}H NMR (500 MHz, D_2O, 25 °C) spectra for (±) **2b**

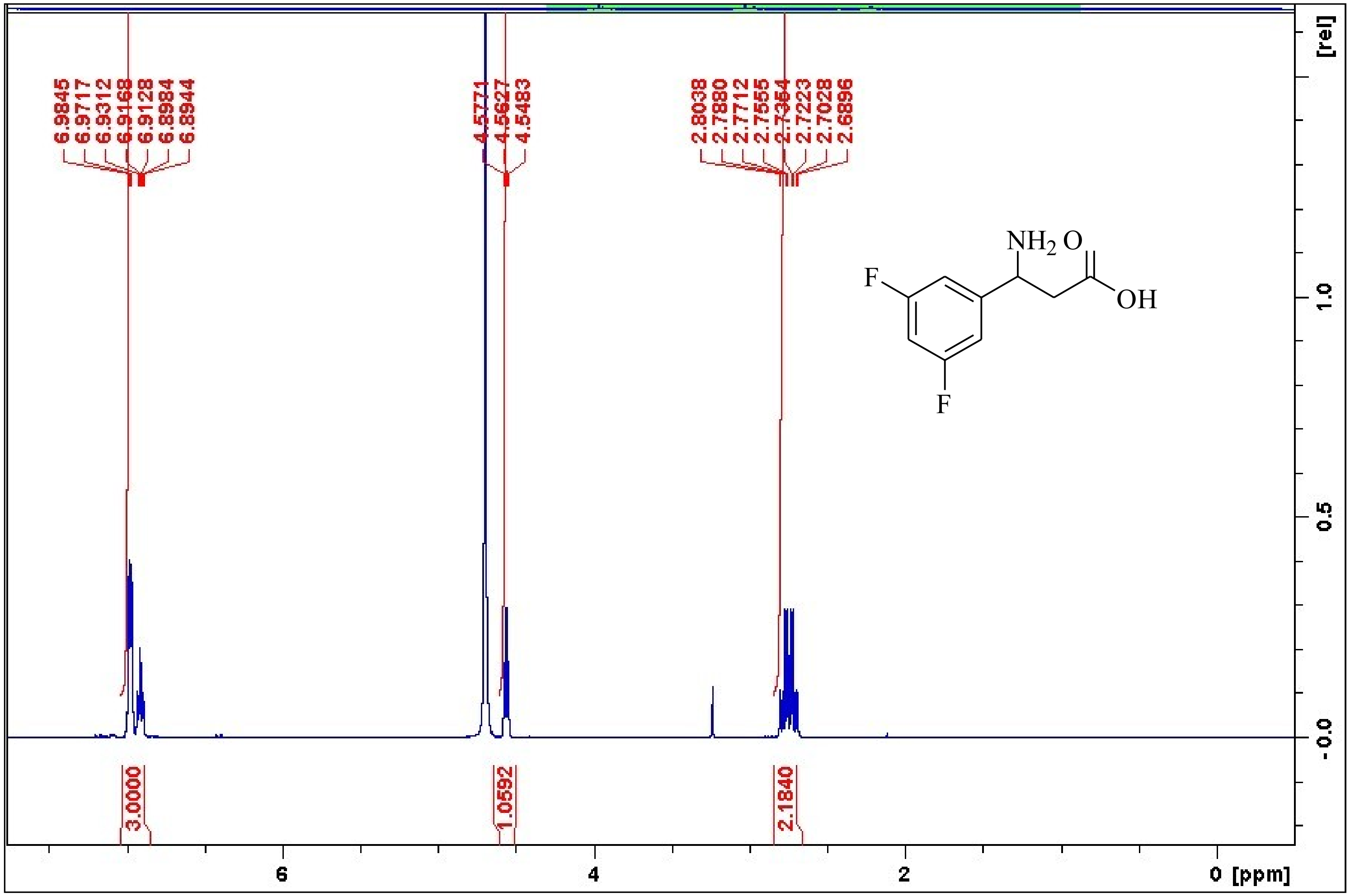

Figure 3. ^{1}H NMR (500 MHz, D_2O, 25 °C) spectra for (±) **2c**

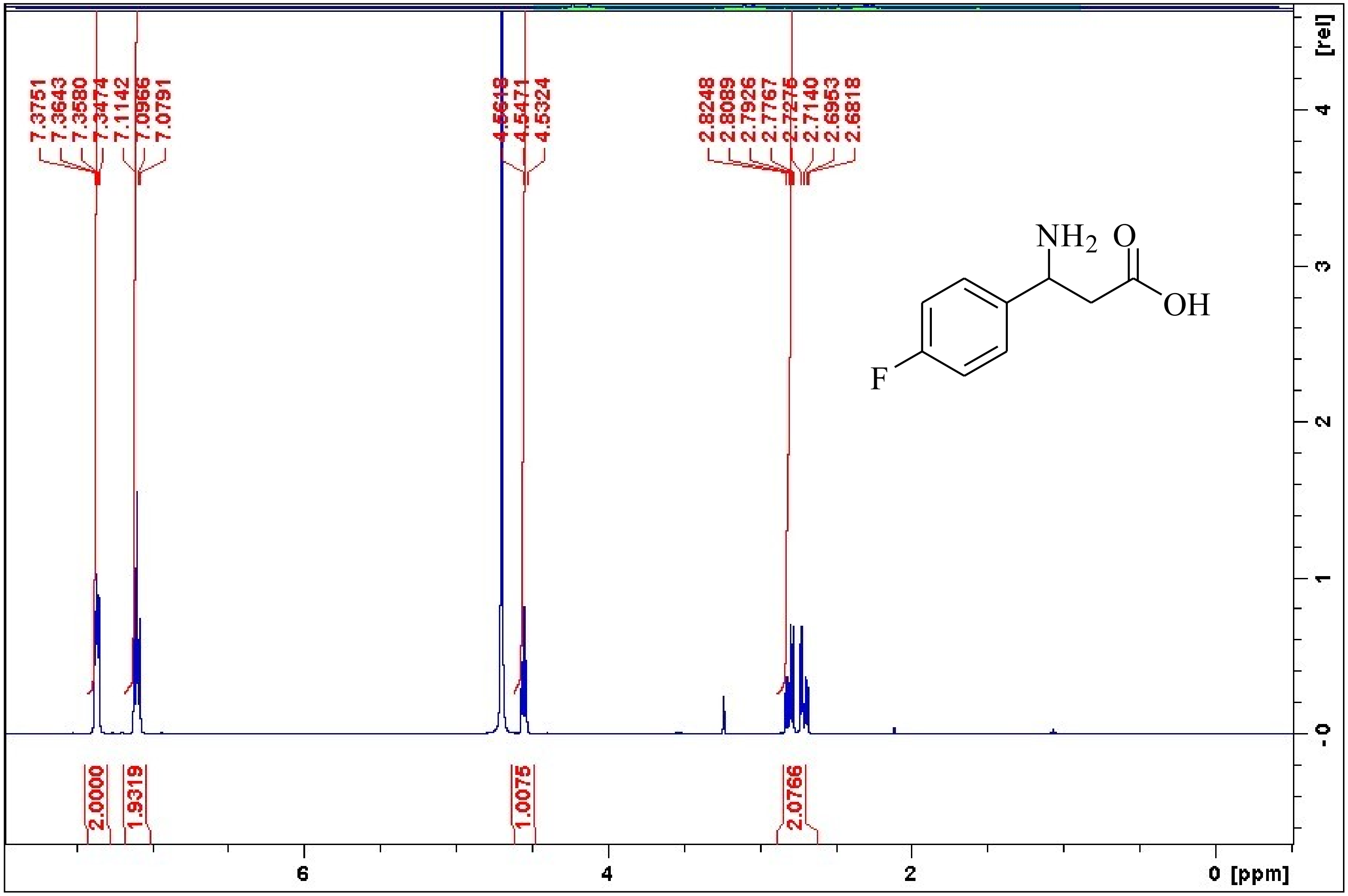

Figure 4. ^1H NMR (500 MHz, D_2O, 25 °C) spectra for (±) **2d**

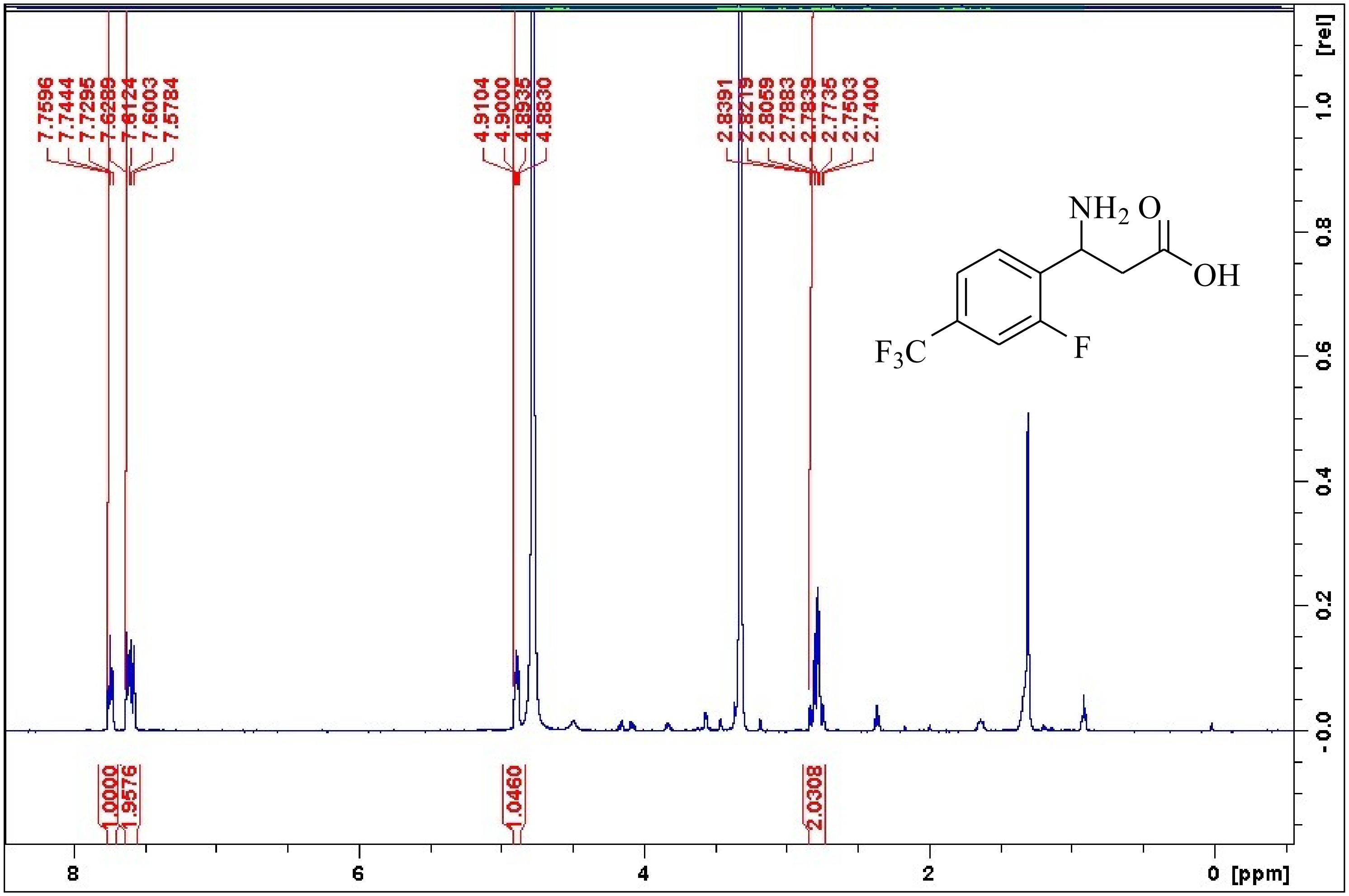

Figure 5. ^{1}H NMR (500 MHz, D_2O, 25 °C) spectra for (±) **2e**

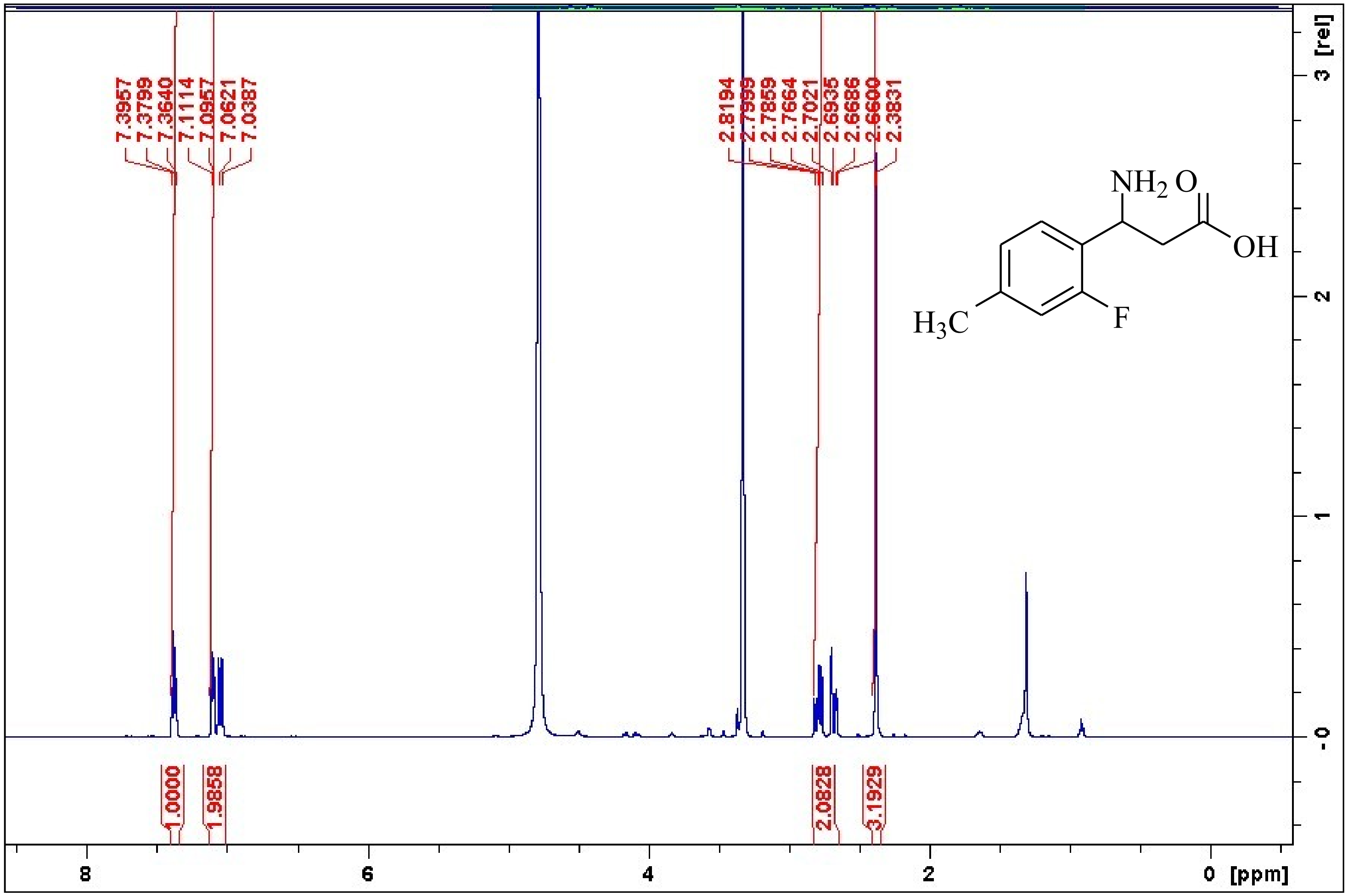

Figure 6. ^{1}H NMR (500 MHz, D_2O, 25 °C) spectra for (±) **3a**

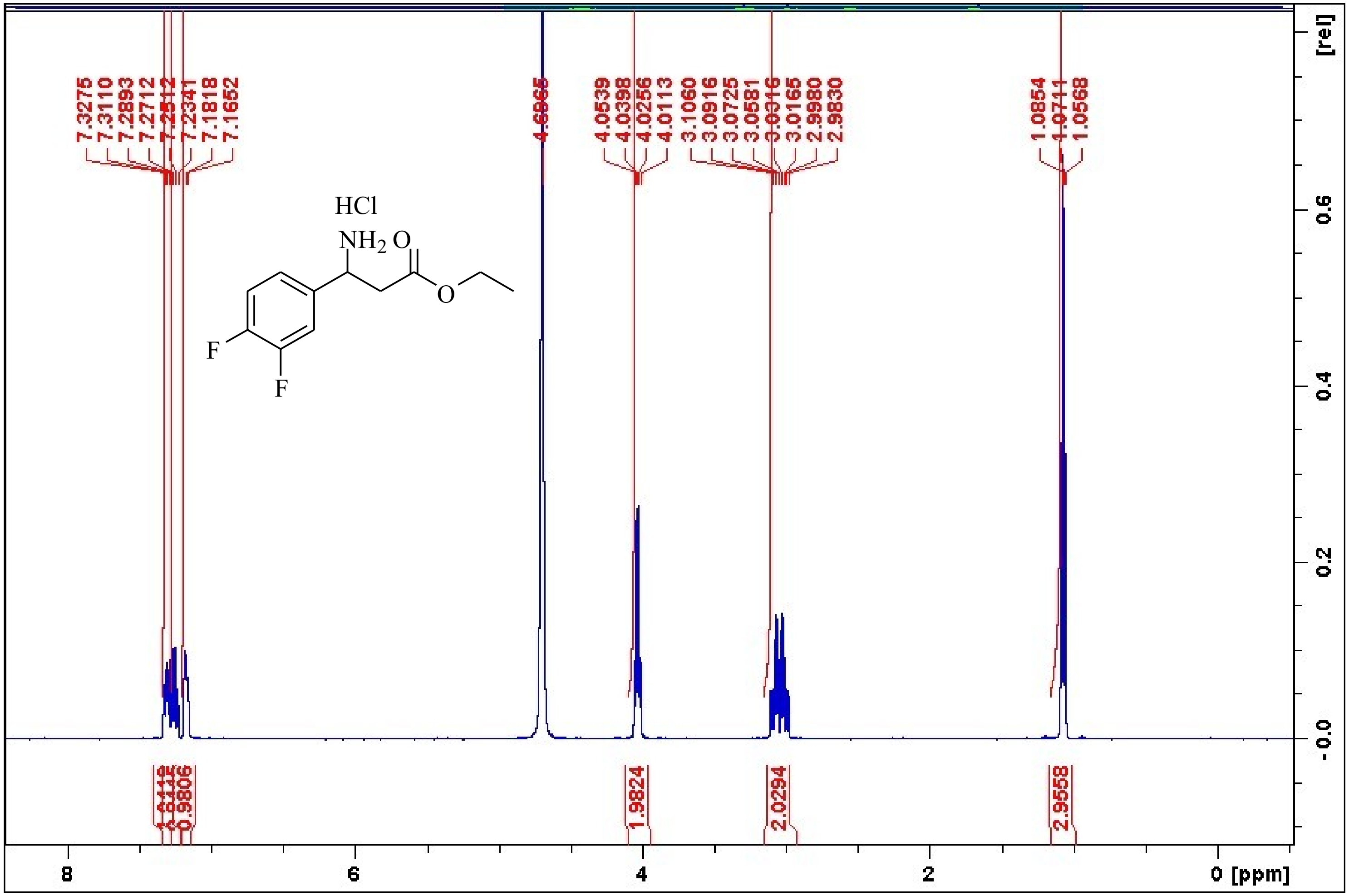

Figure 7. ^{1}H NMR (500 MHz, D_2O, 25 °C) spectra for (±) **3b**

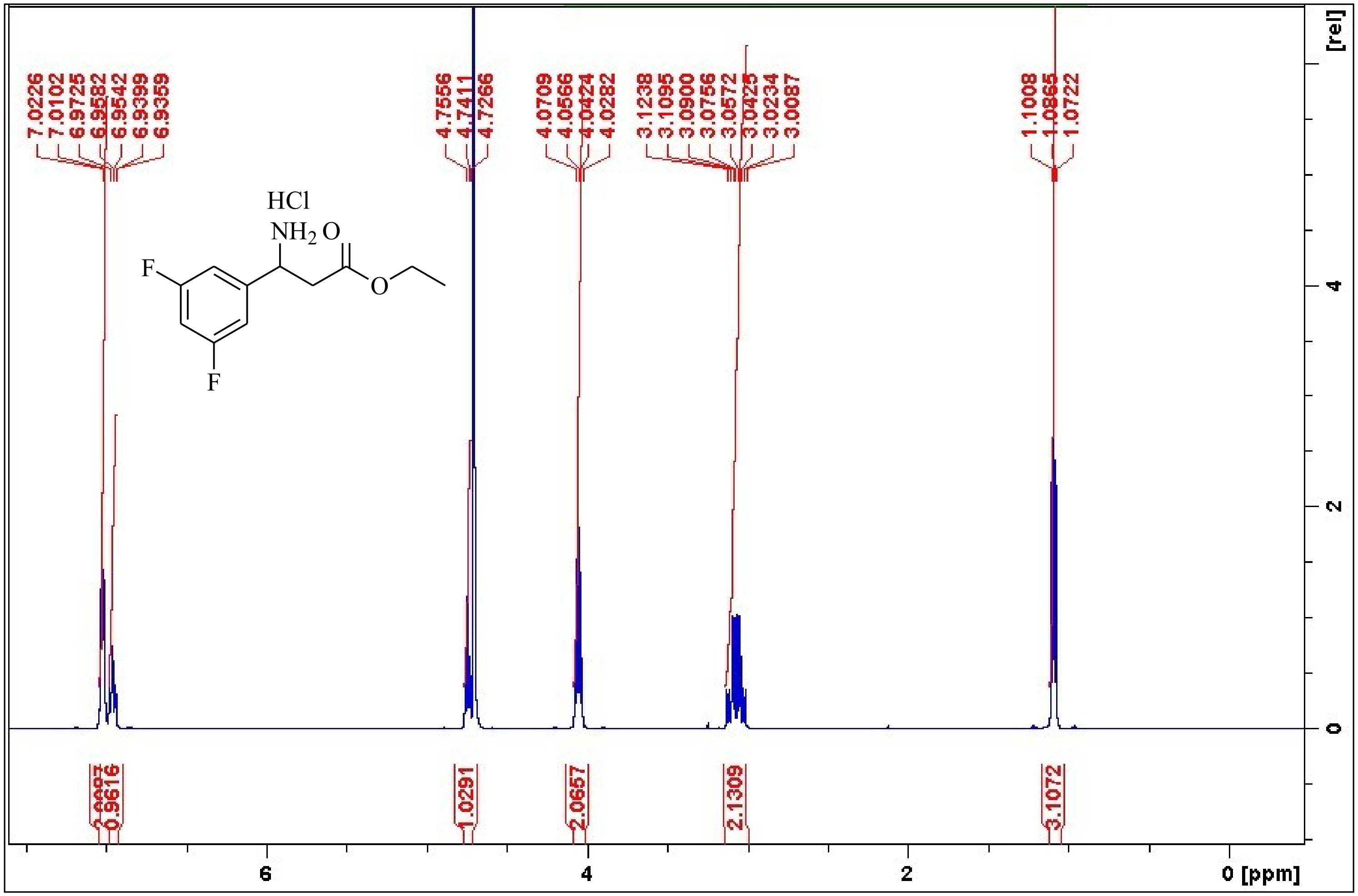

Figure 8. ^{1}H NMR (500 MHz, D_2O, 25 °C) spectra for (±) **3c**

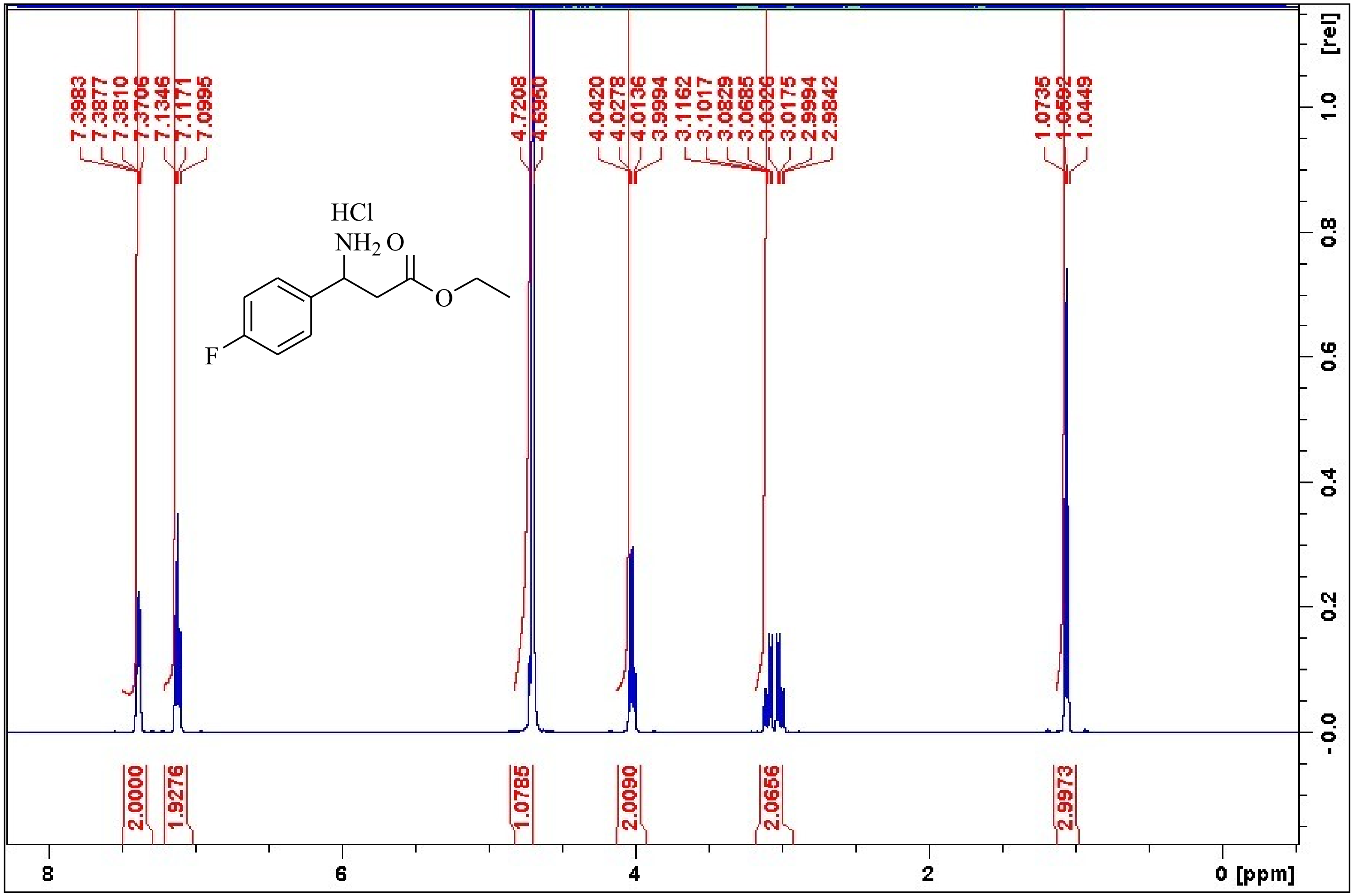

Figure 9. ^{1}H NMR (500 MHz, D_2O, 25 °C) spectra for (±) **3d**

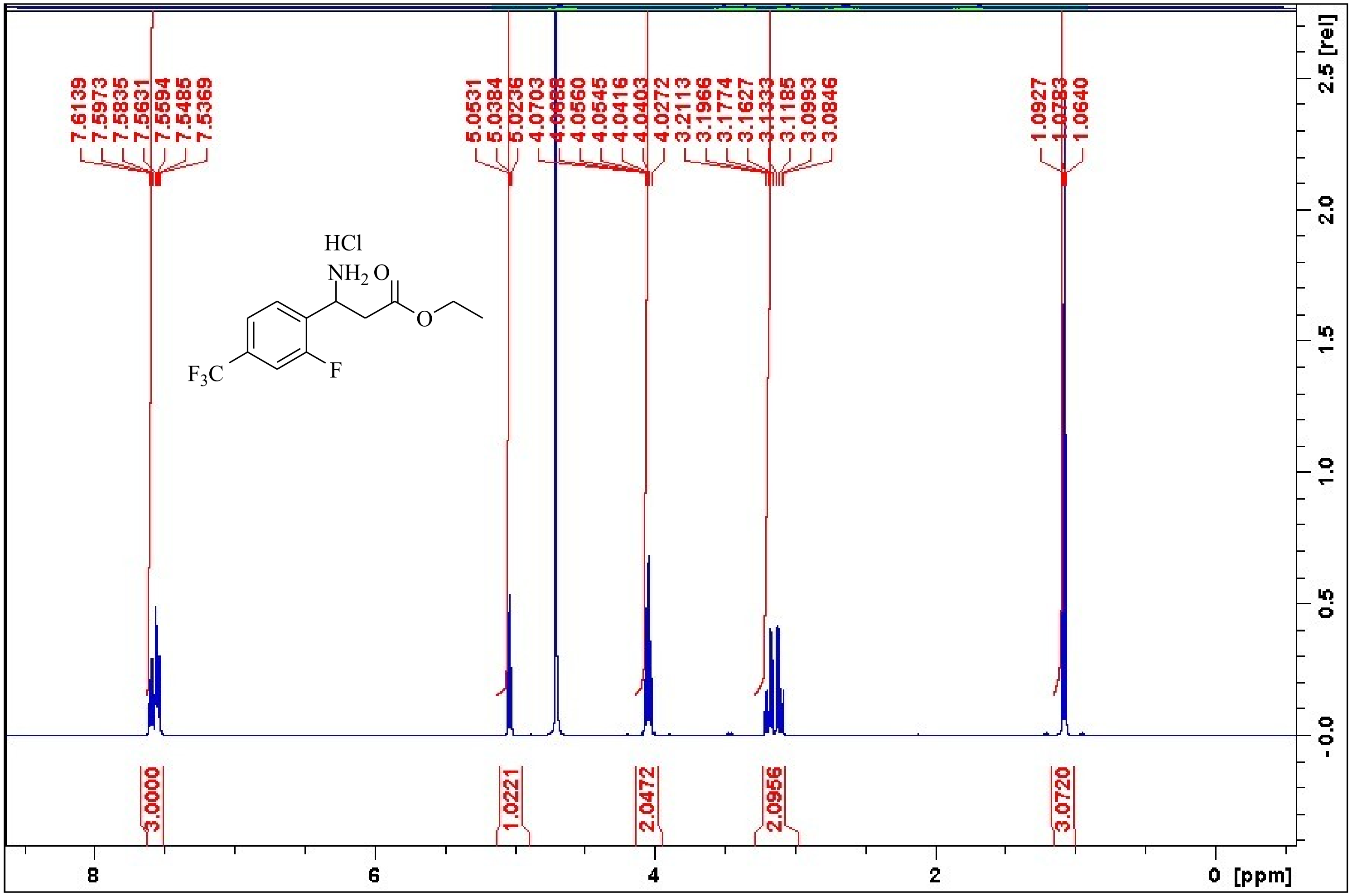

Figure 10. ^{1}H NMR (500 MHz, D_2O, 25 °C) spectra for (±) **3e**

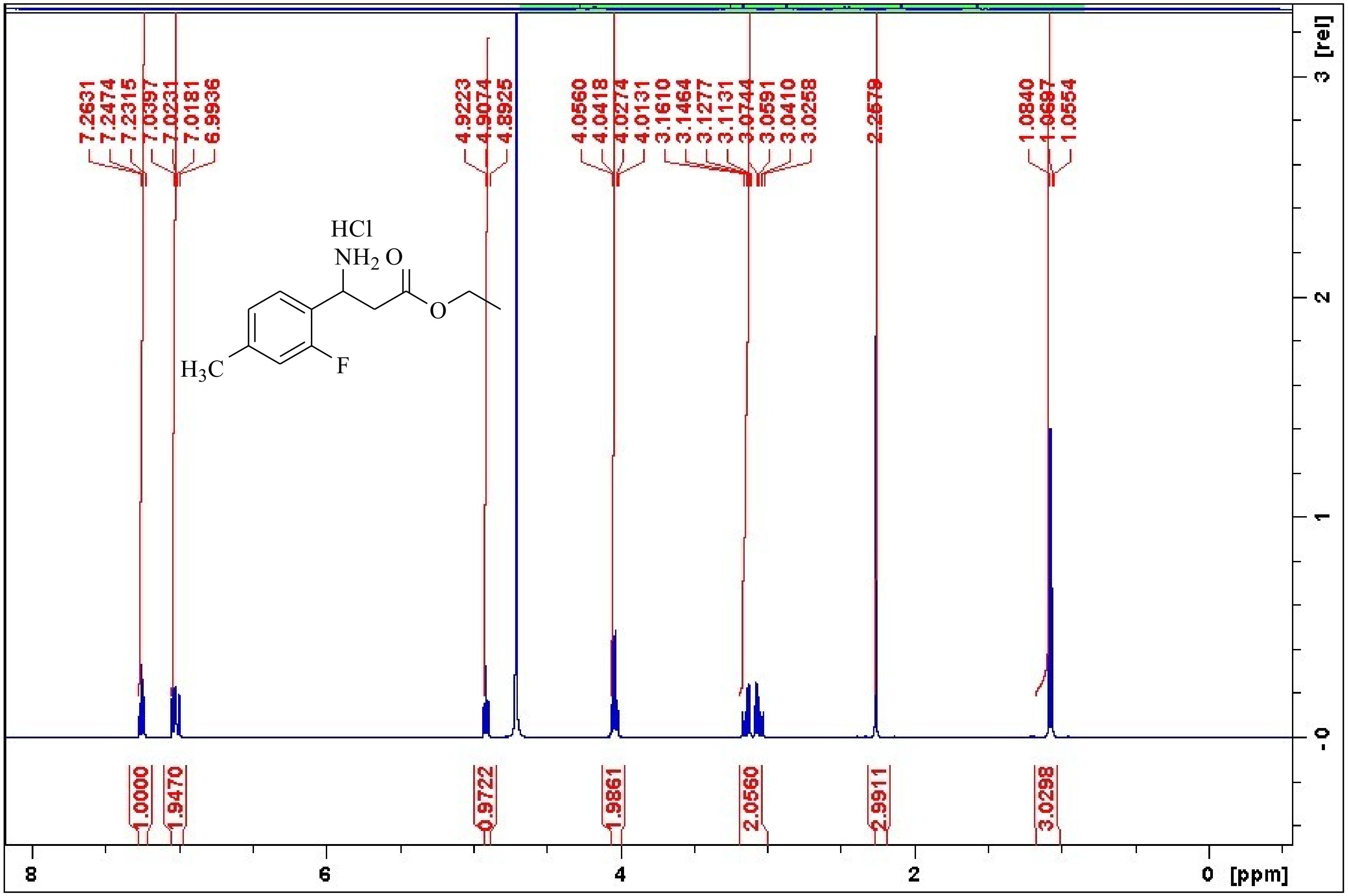

Figure 11. ^1H NMR (500 MHz, D_2O, 25 °C) spectra for (*R*) **4a** and (*S*) **5a**

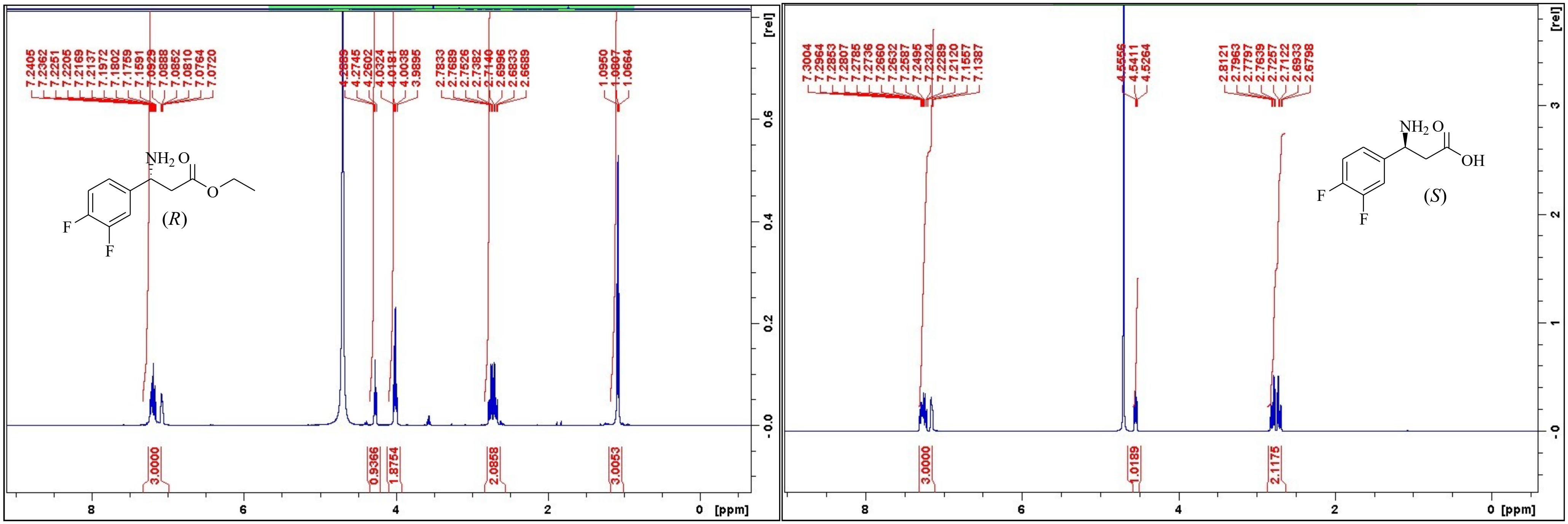

Figure 12. ¹H NMR (500 MHz, D_2O, 25 °C) spectra for (*R*) **4b** and (*S*) **5b**

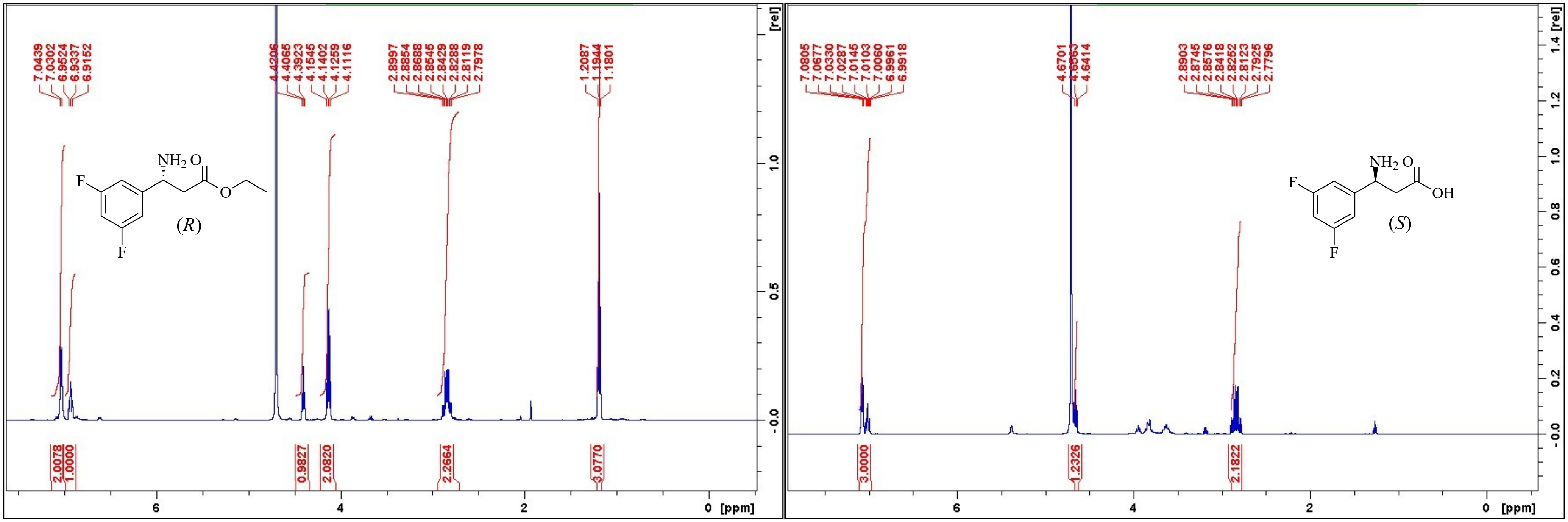

Figure 13. ^{1}H NMR (500 MHz, D_2O, 25 °C) spectra for (*R*) **4c** and (*S*) **5c**

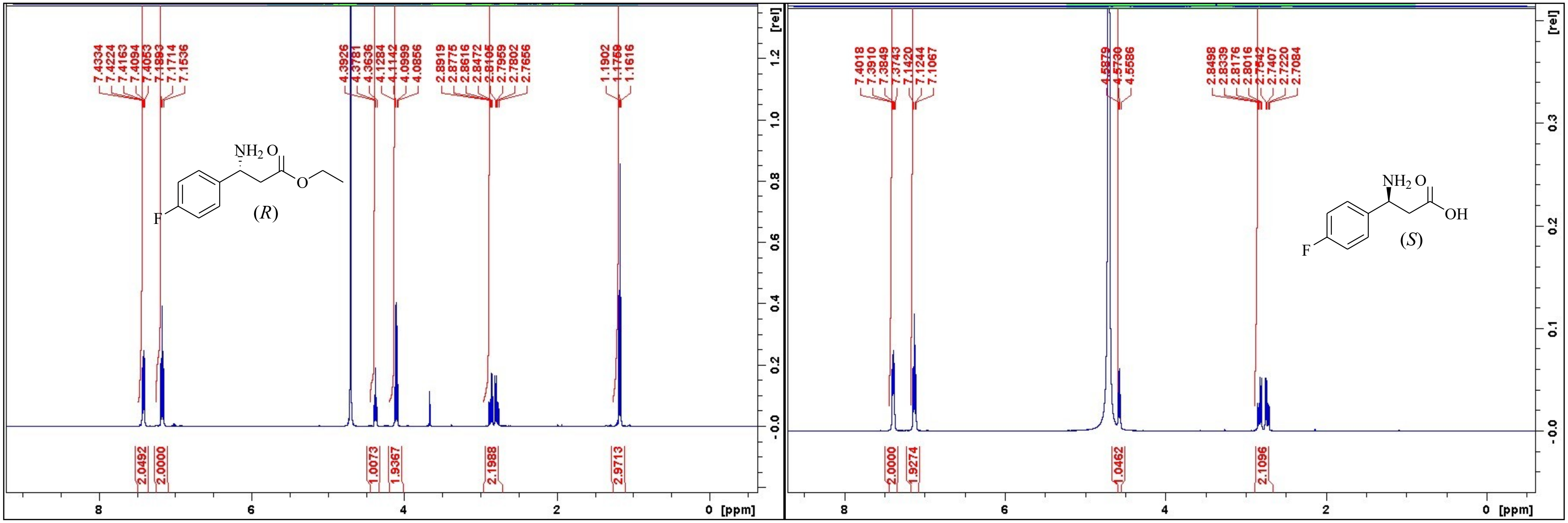

Figure 14. ^{1}H NMR (500 MHz, D_2O, 25 °C) spectra for (*R*) **4d** and (*S*) **5d**

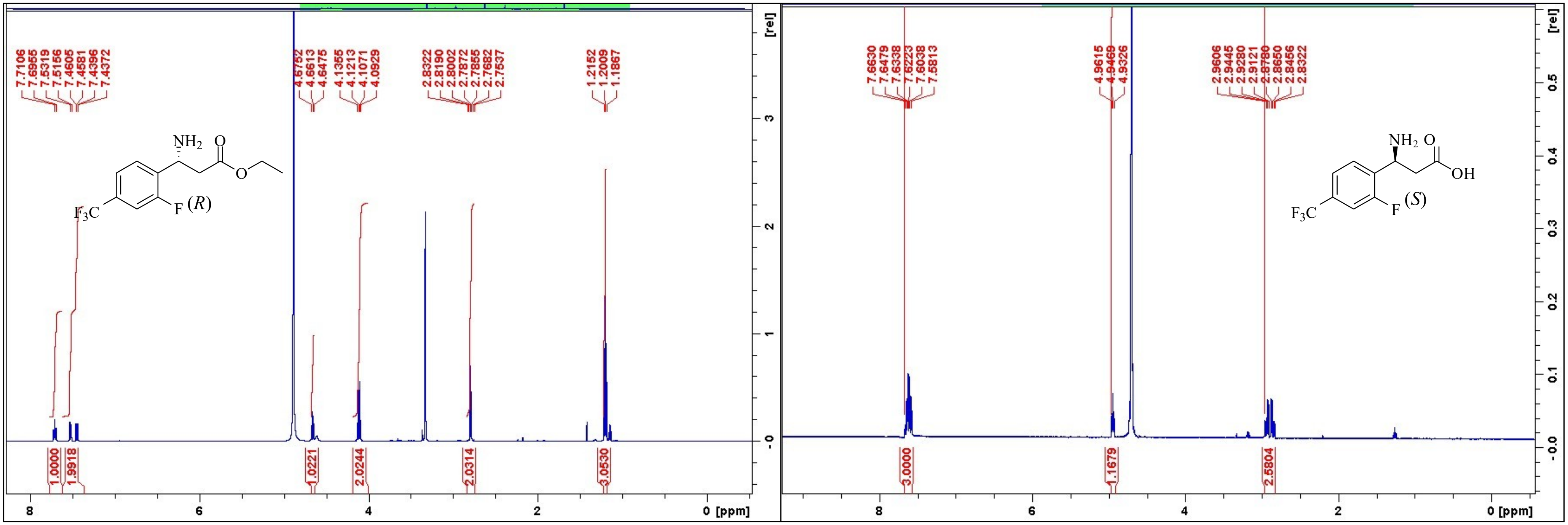

Figure 15. ^{1}H NMR (500 MHz, D_2O, 25 °C) spectra for (*R*) **4e** and (*S*) **5e**

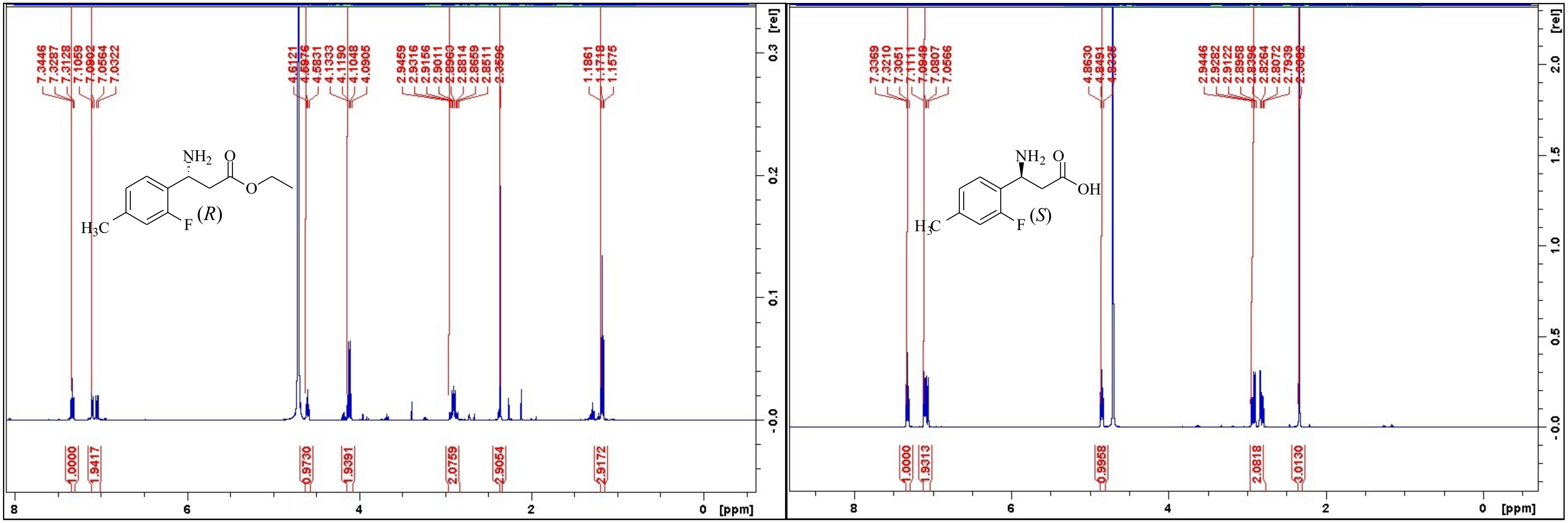

GC equipped with a Chirasil-L-Val column (25m, 0.25mm, 0.12μm)
90 °C for 10 min → 170 °C (temperature rise 20 °C min^{-1}), 10 psi

Figure 16. Retention times (min) for **5a**: 32.365 (antipode:31.515)

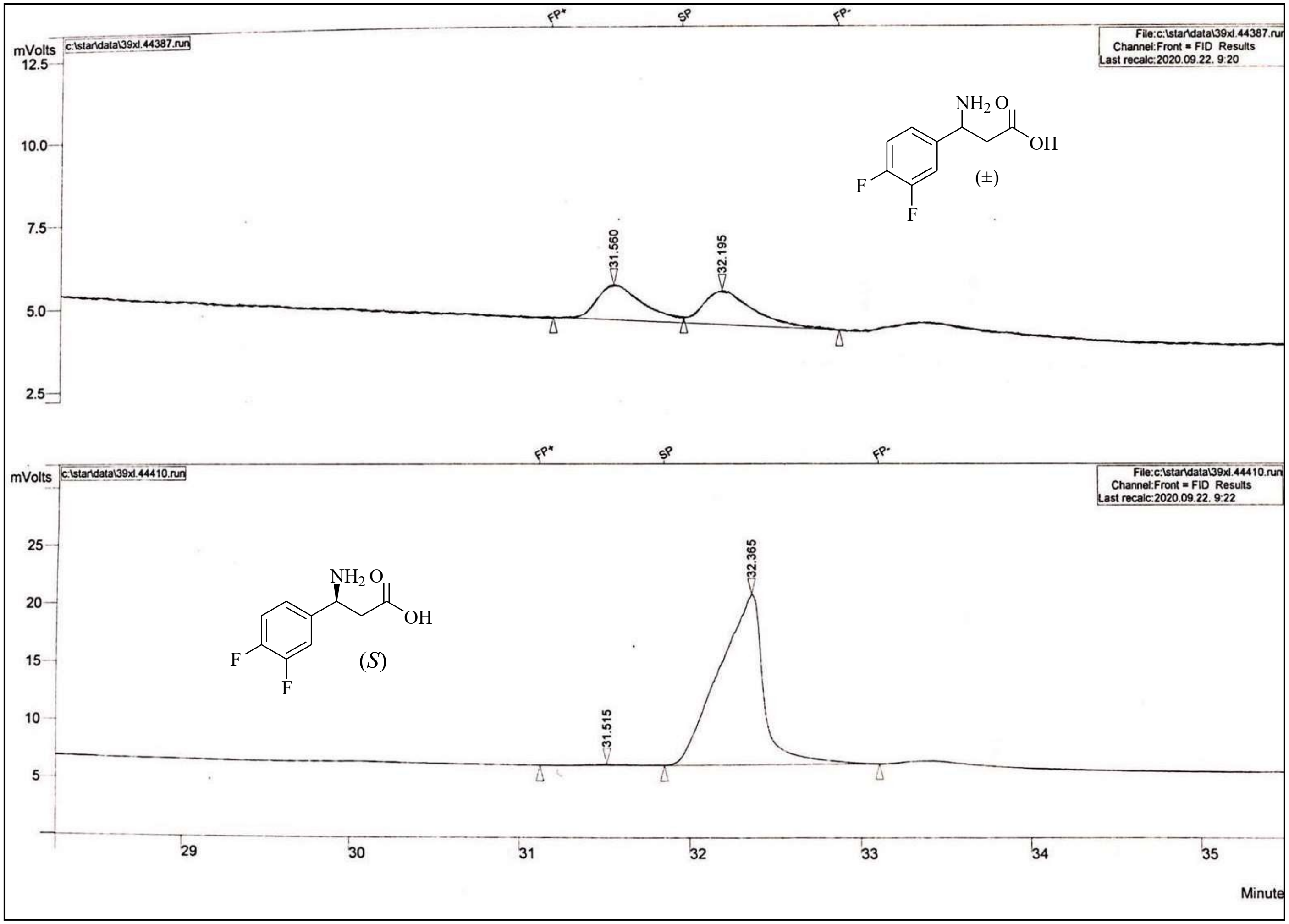

Figure 17. Retention time for **4a**: 36.308 (antipode: 36.535)

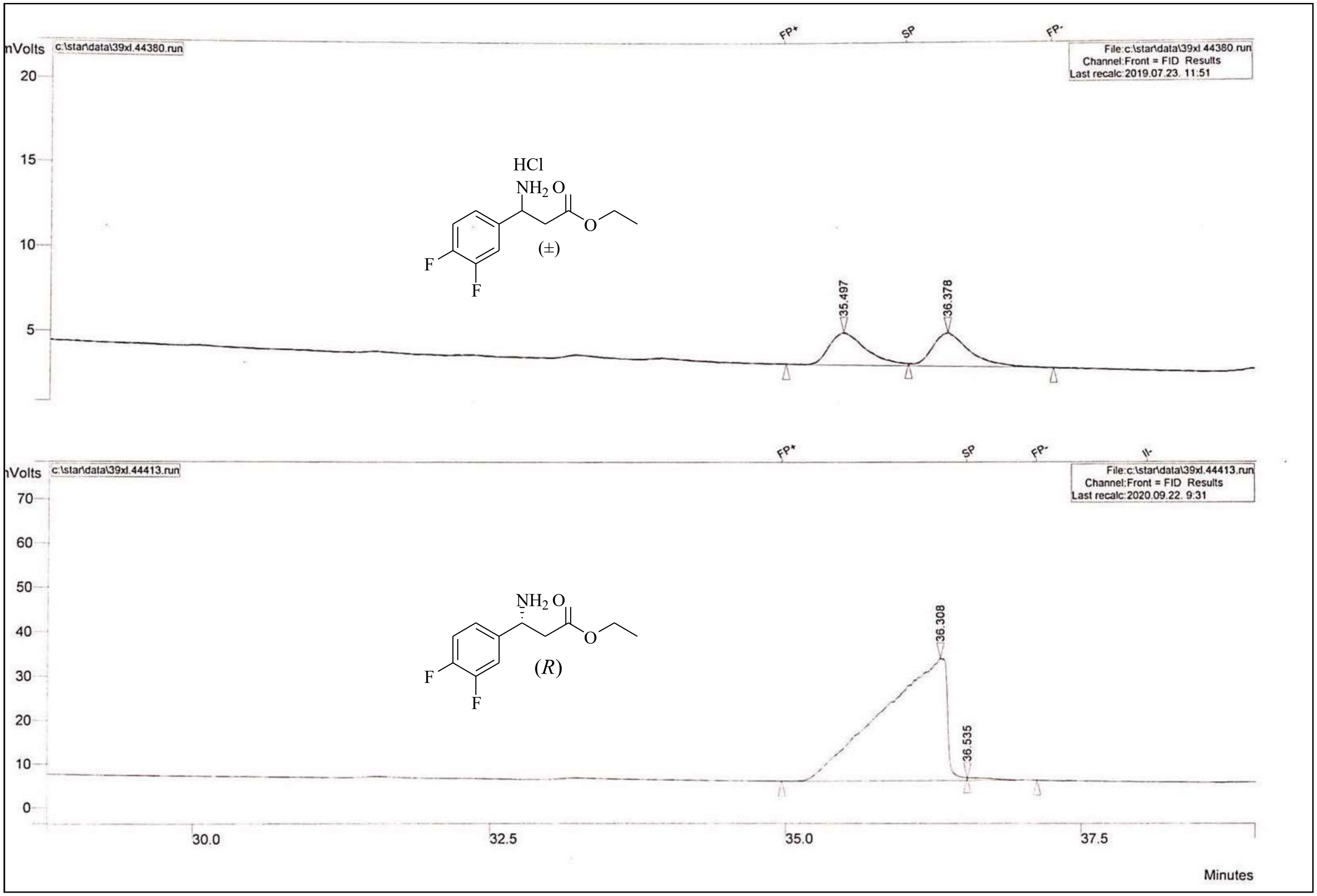

Figure 18. Retention time for **5b**: 29.031 (antipode: 28.282)

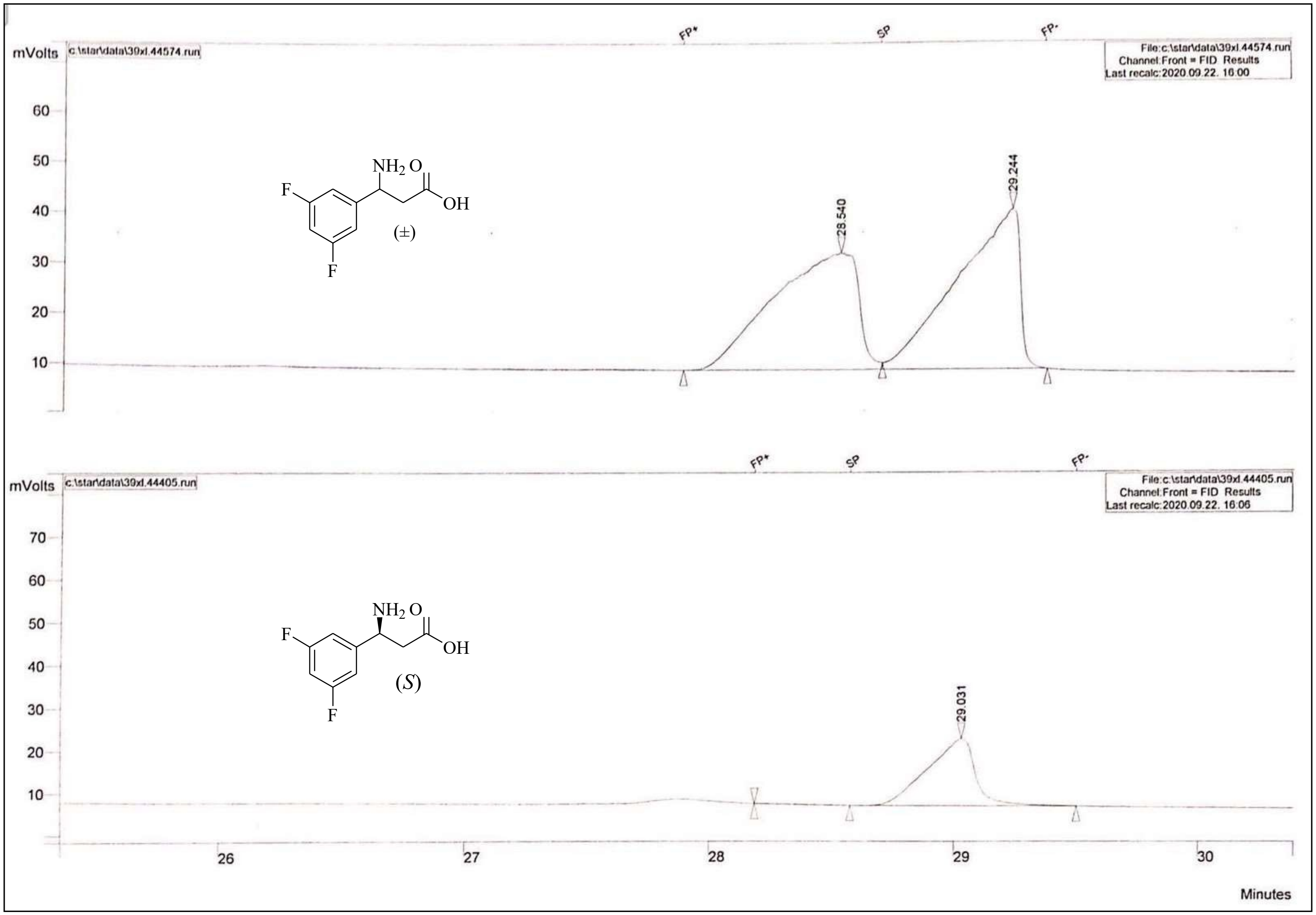

Figure 19. Retention times for **4b**: 32.137 (antipode: 32.550)

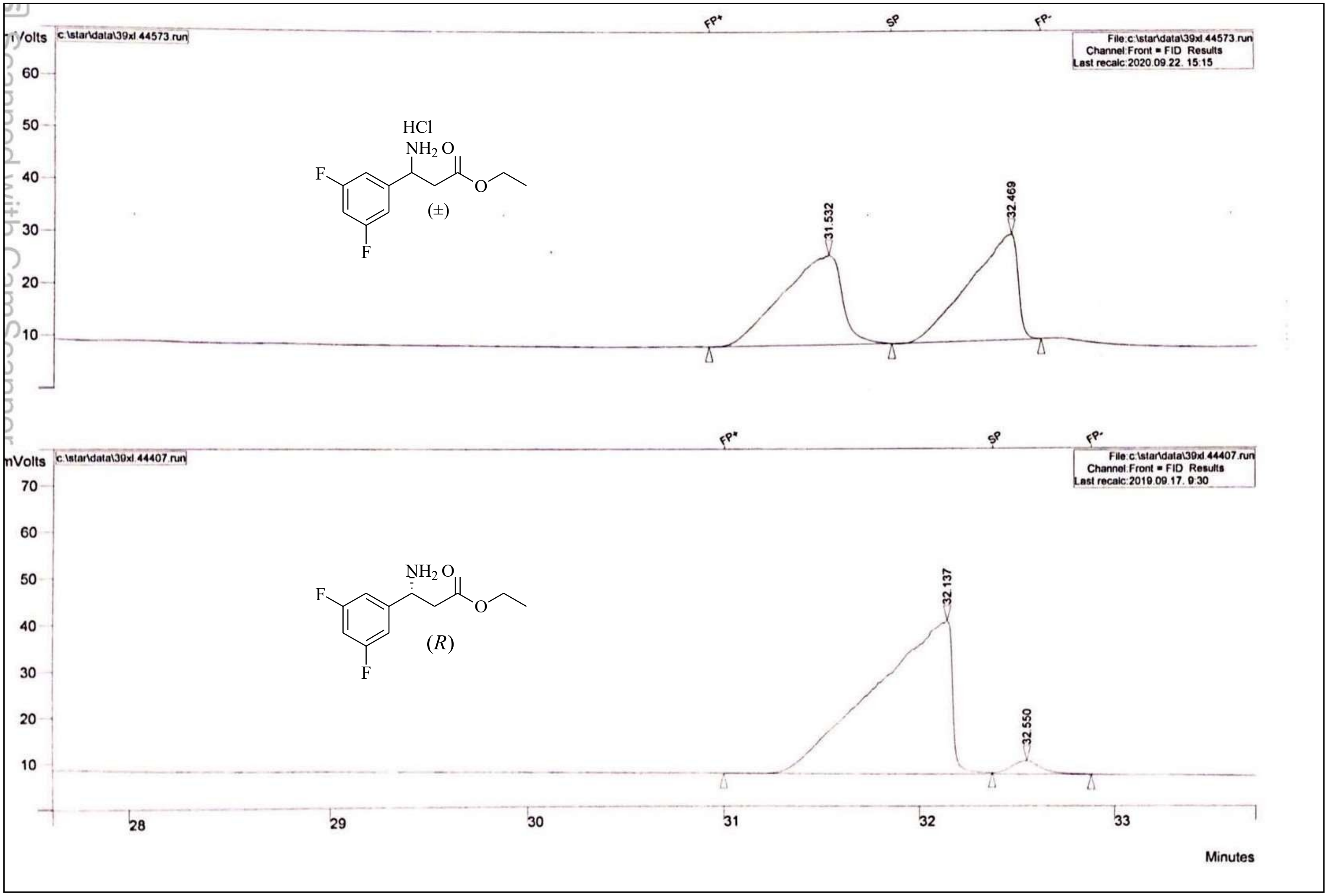

Figure 20. Retention times for **5c**: 29.905 (antipode: 29.528)

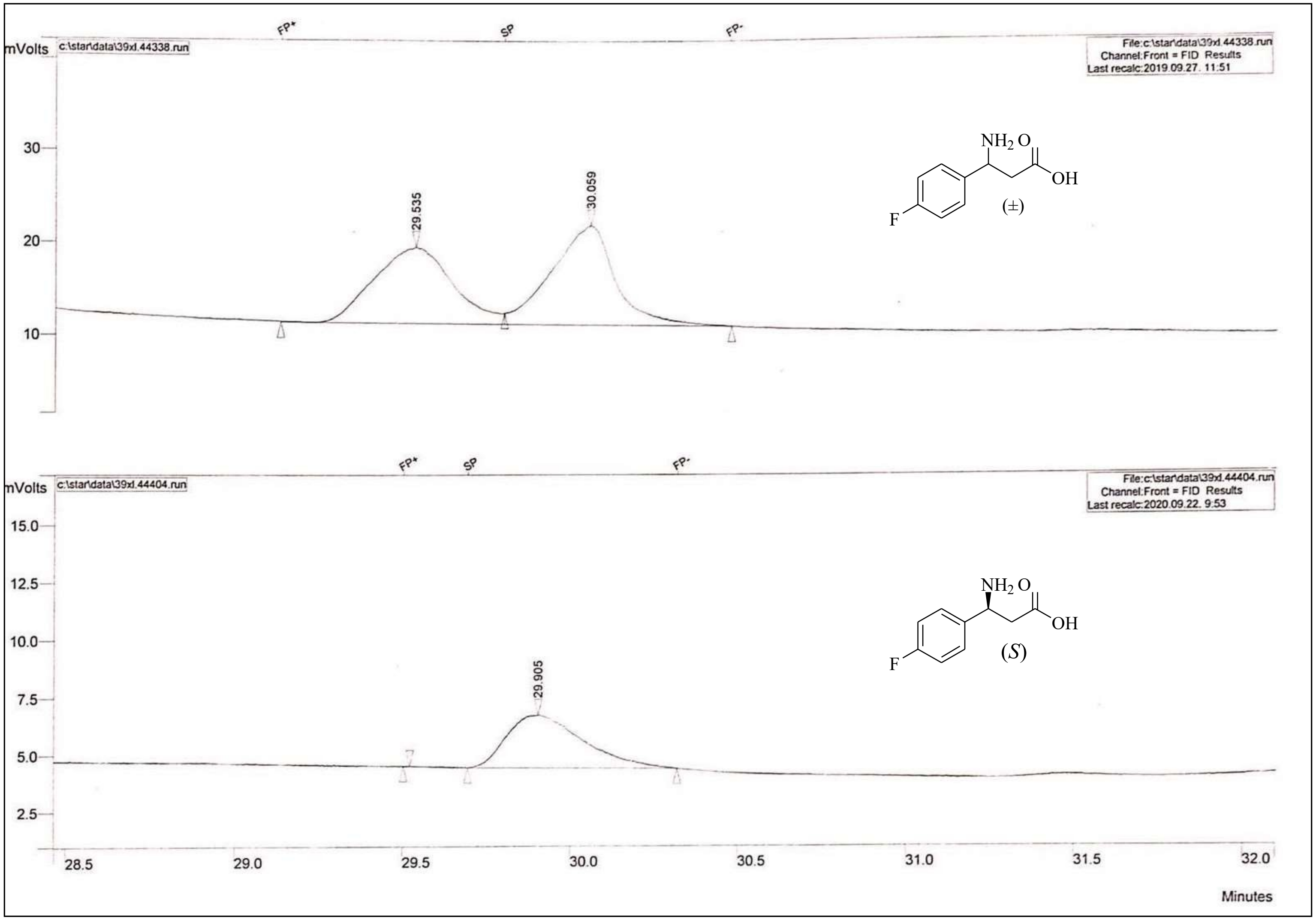

Figure 21. Retention times for **4c**: 33.305 (antipode: 33.860)

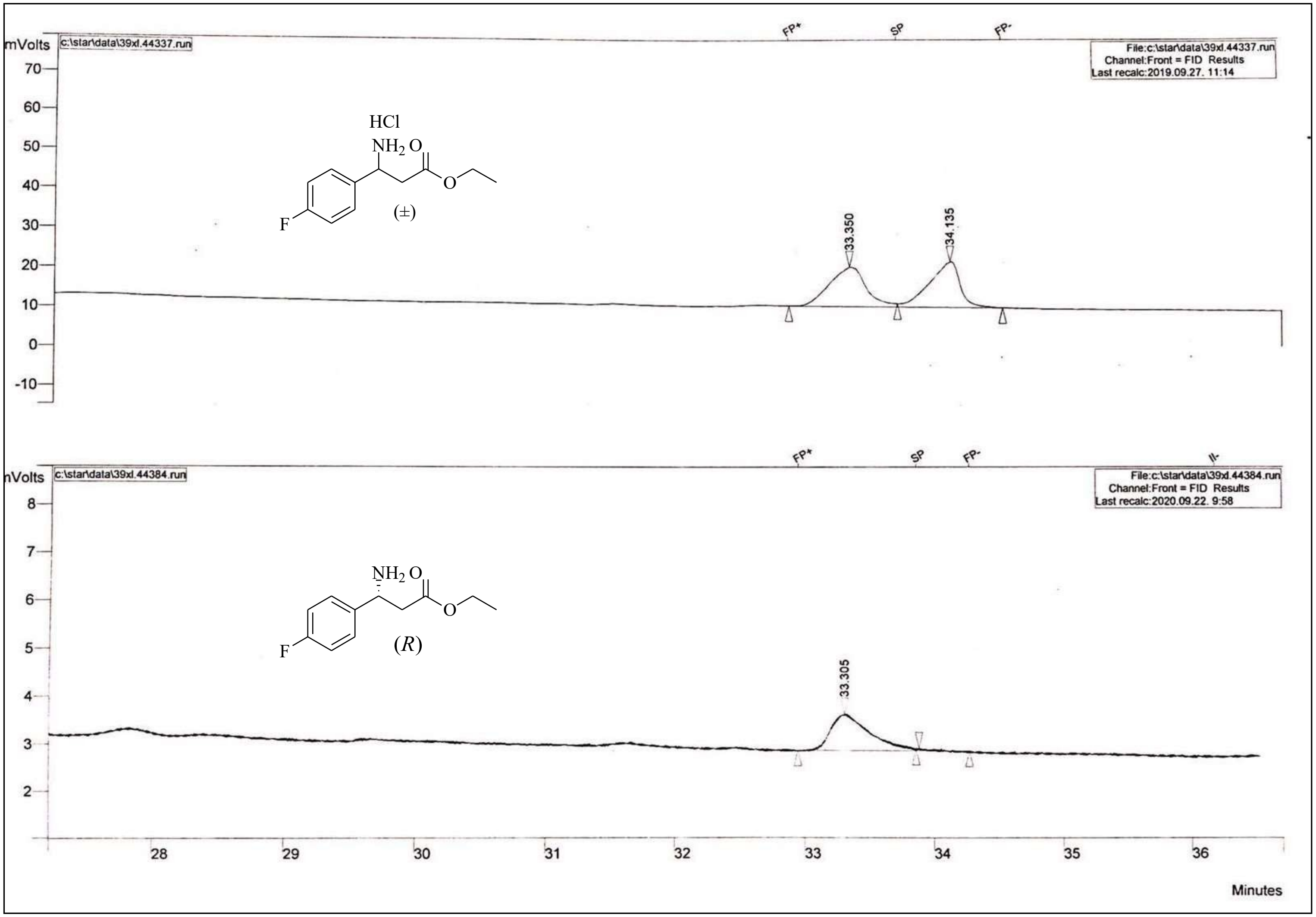

Figure 22. Retention times for **5d**: 23.766 (antipode: 23.463)

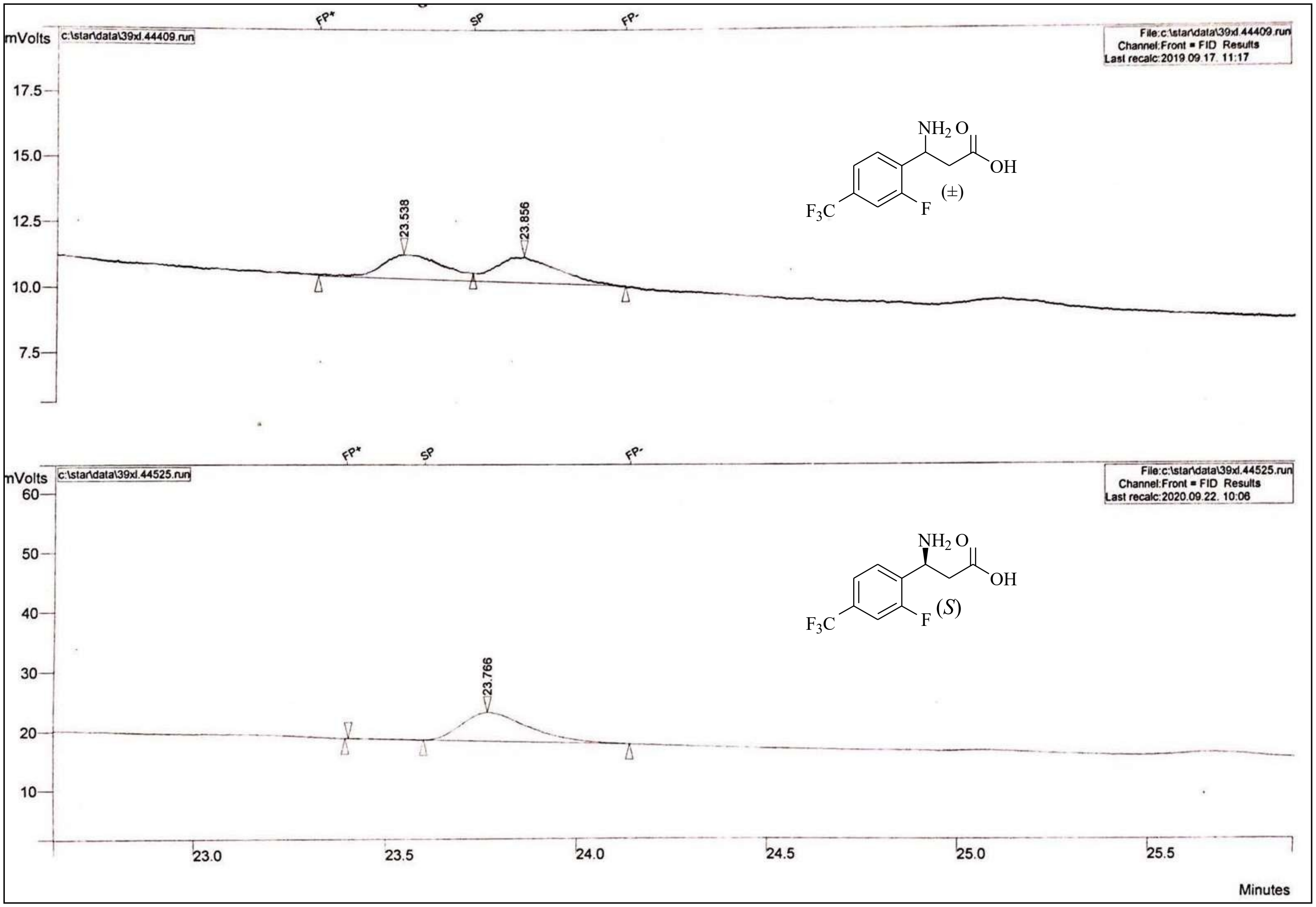

Figure 23. Retention times for **4d**: 26.064 (antipode: 26.187)

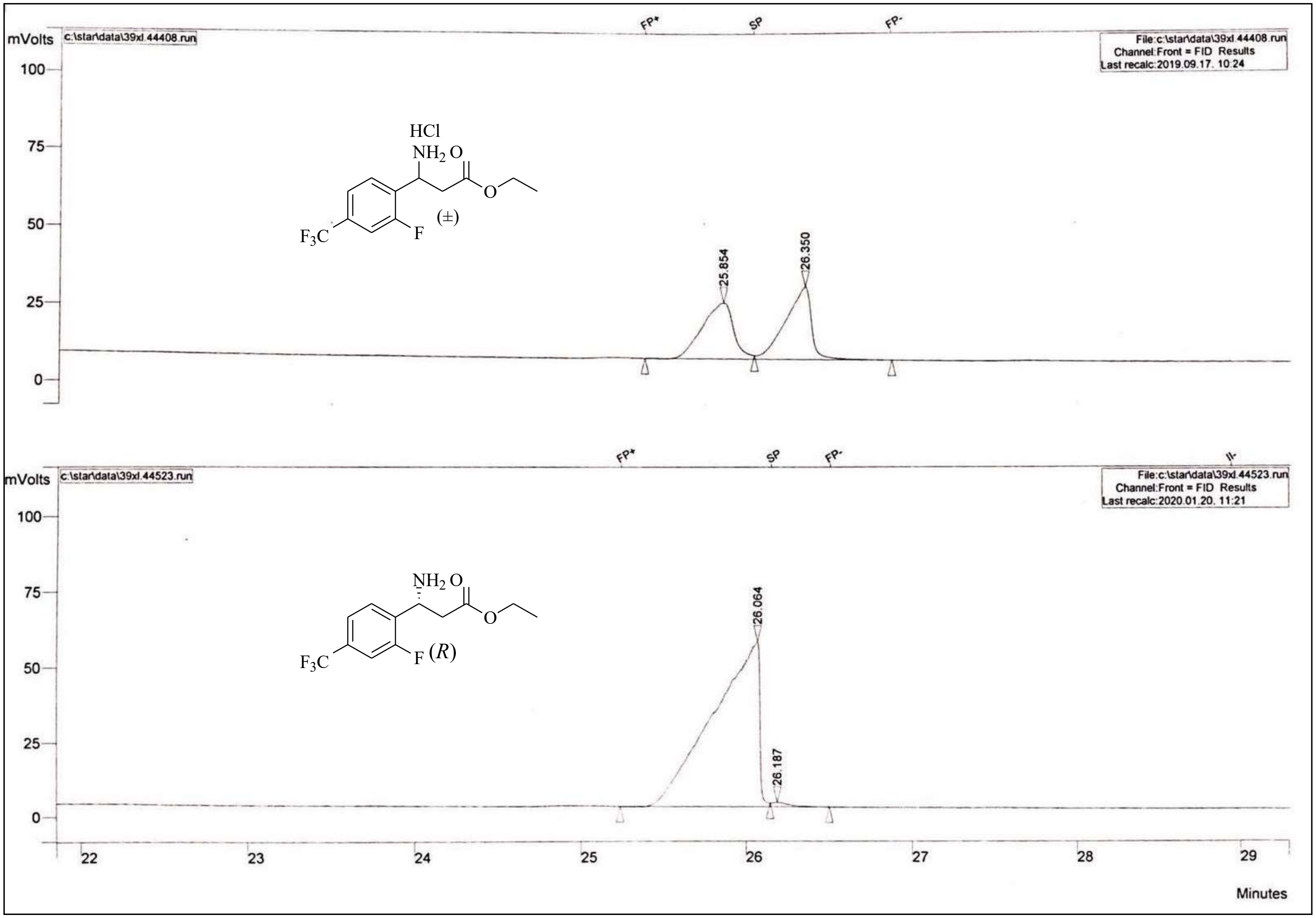

Figure 24. Retention times for **5e**: 30.946 (antipode: 30.541)

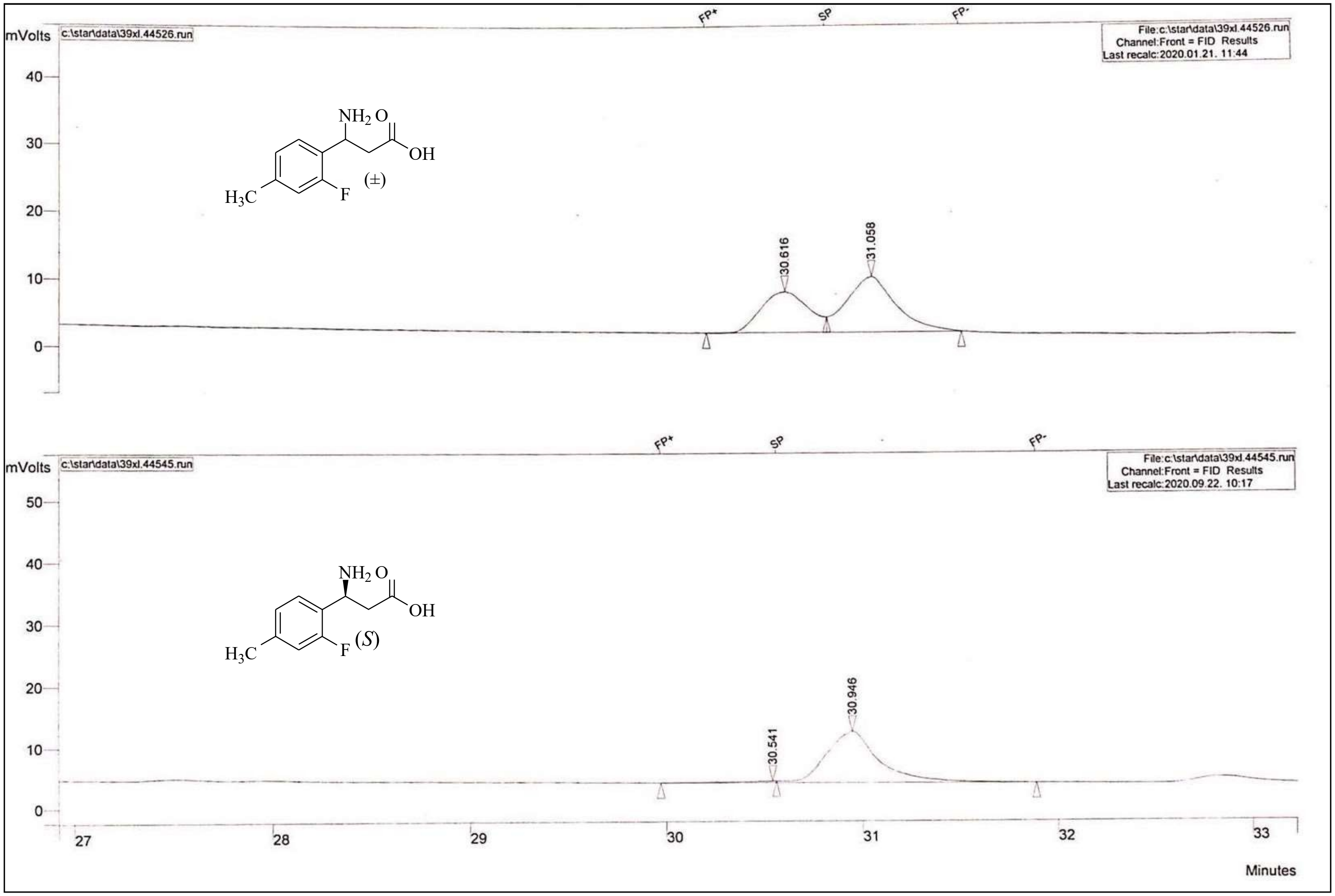

Figure 25. Retention times for **4e**: 35.421 (antipode: 36.018)

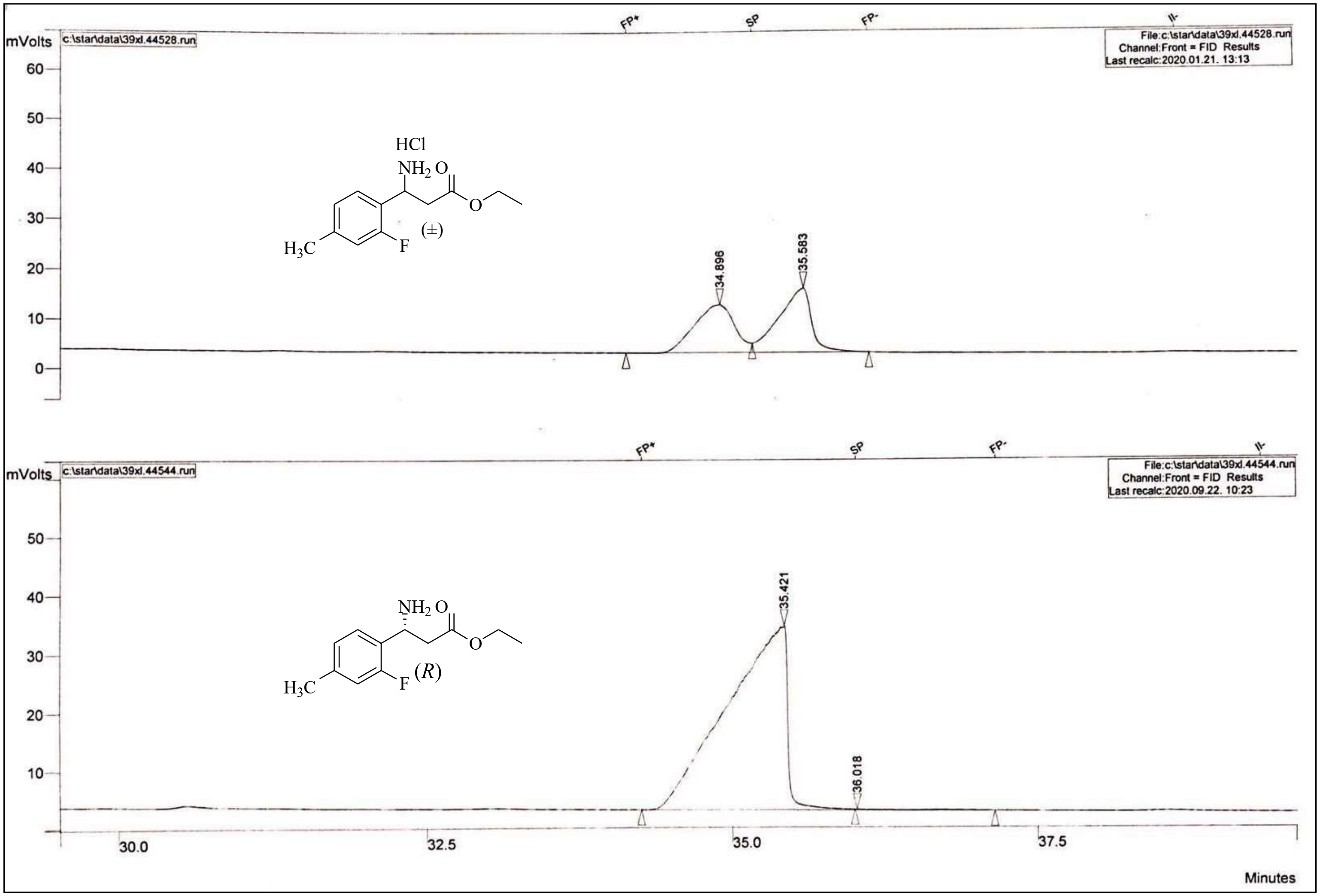

Figure 26. ^{13}C NMR (126 MHz, D_2O) spectra for (±) **3a**

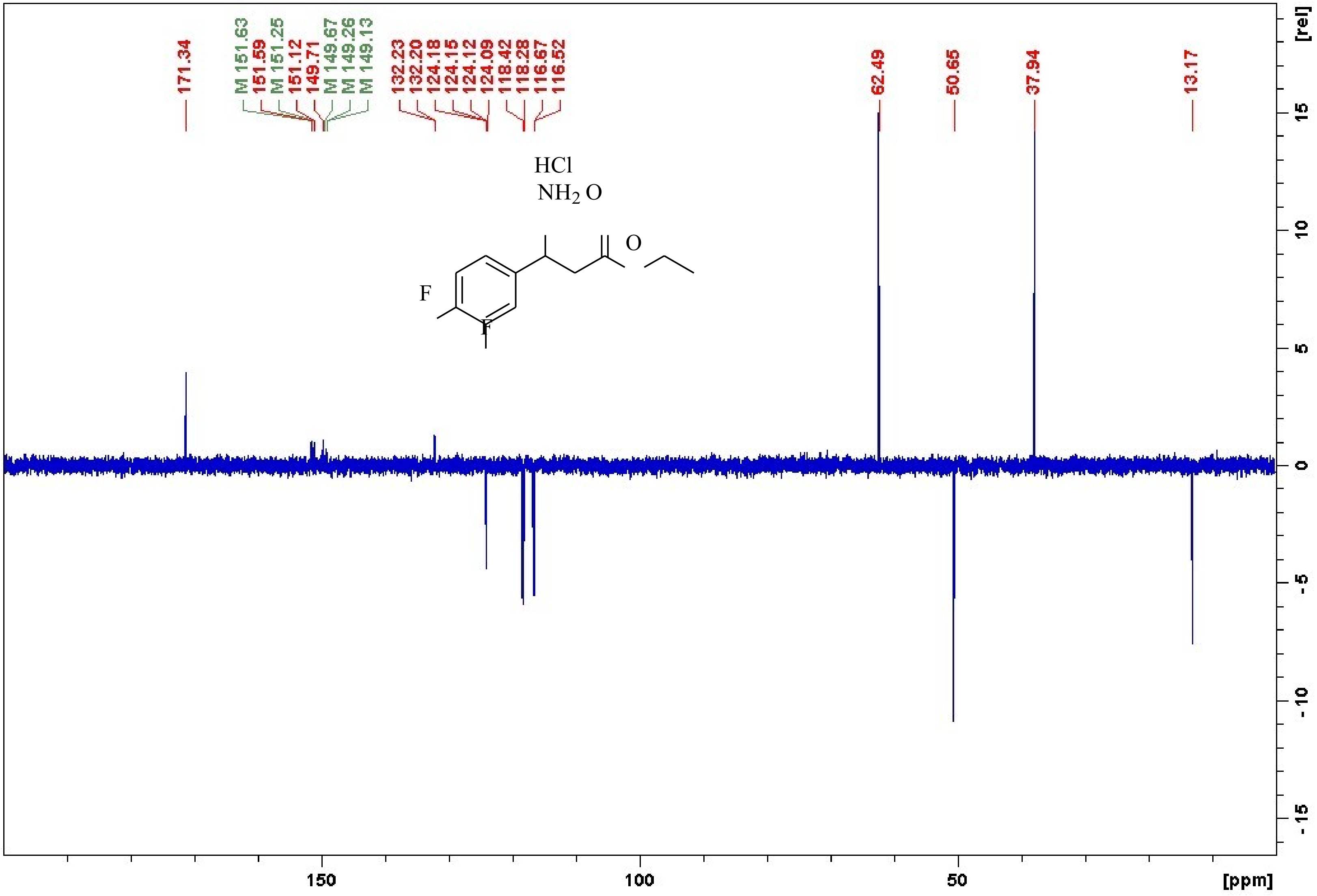

Figure 27. ^{13}C NMR (126 MHz, D_2O) spectra for (±) **3b**

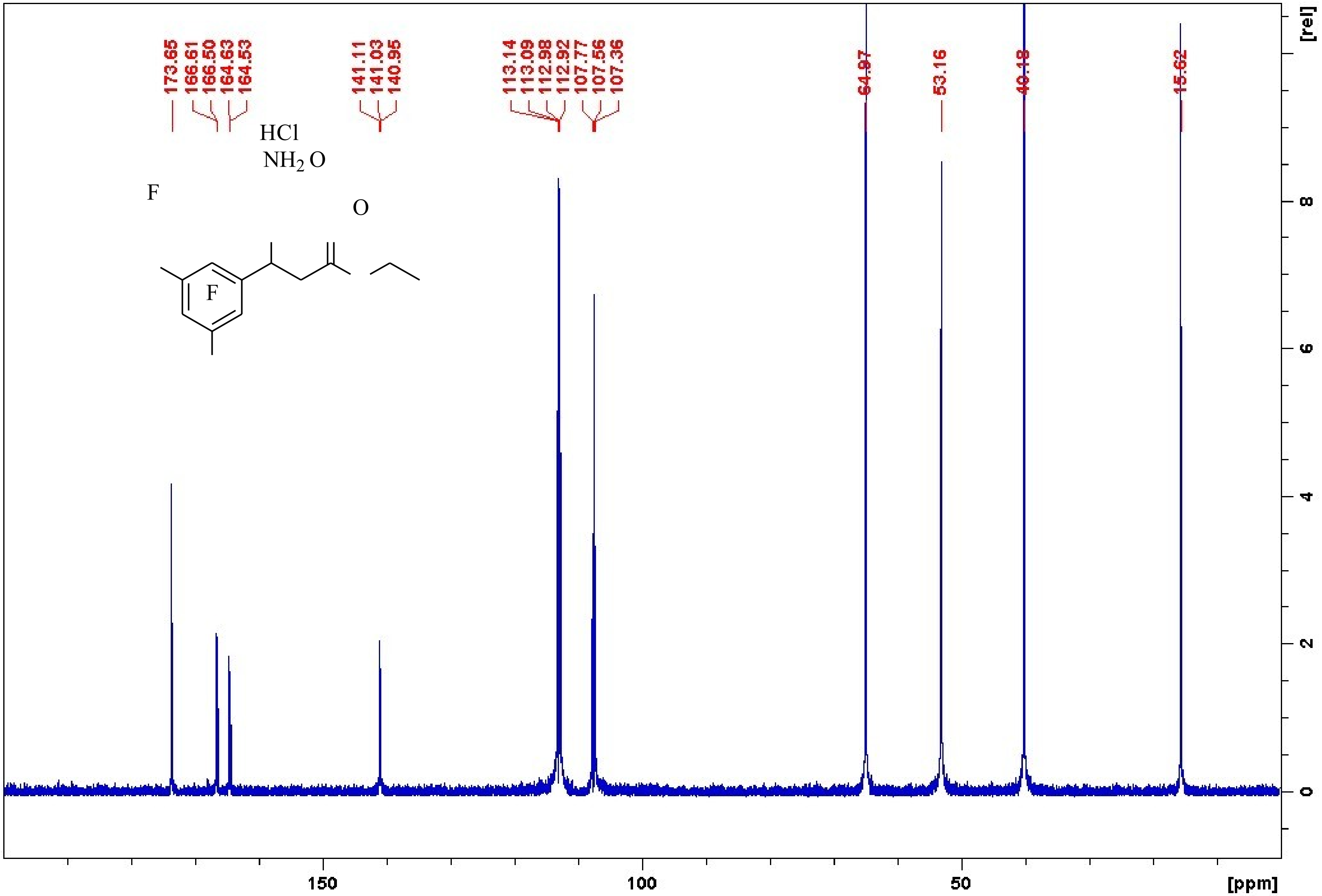

Figure 28. ^{13}C NMR (126 MHz, D_2O) spectra for (±) **3c**

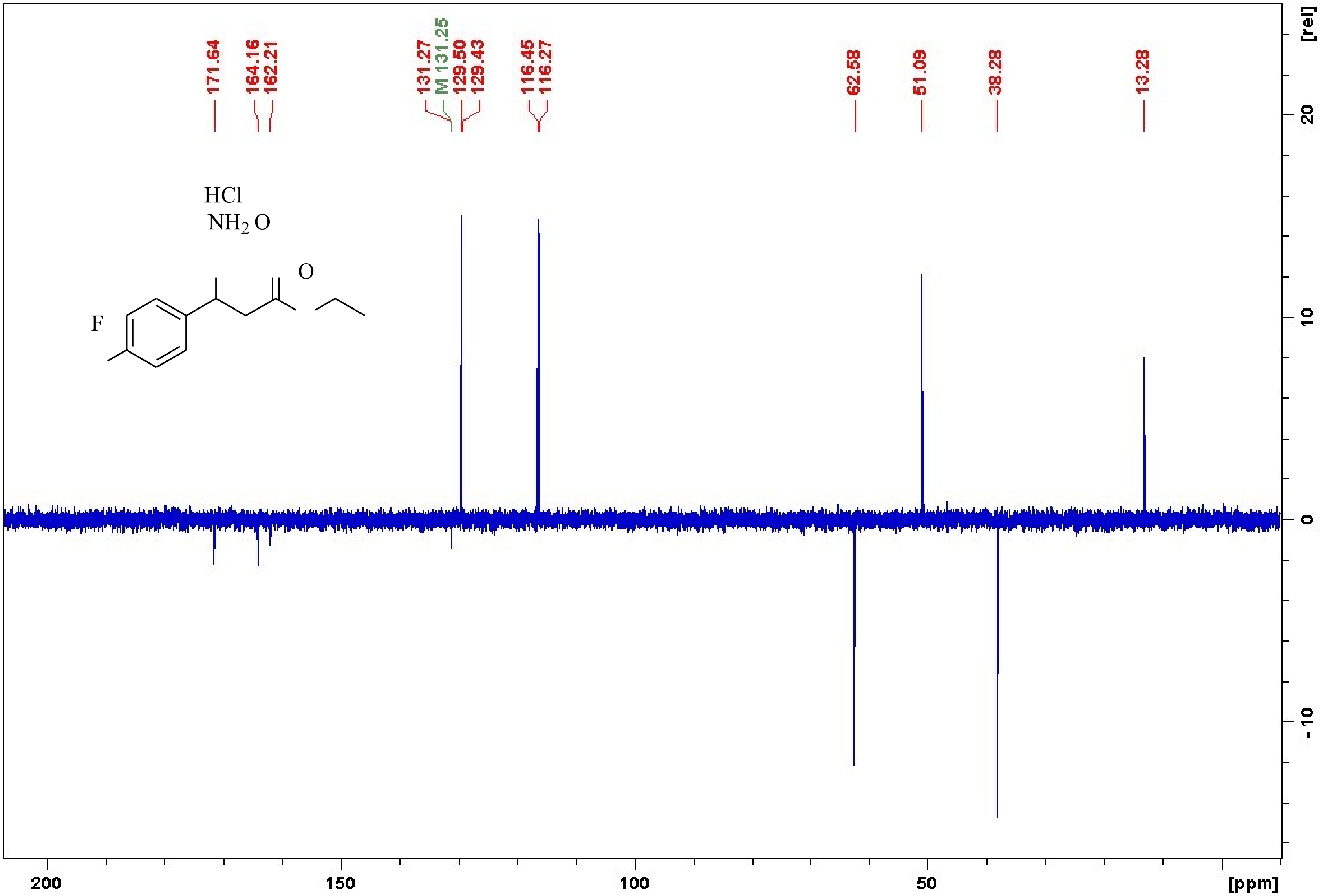

Figure 29. ^{13}C NMR (126 MHz, D_2O) spectra for (±) **3d**

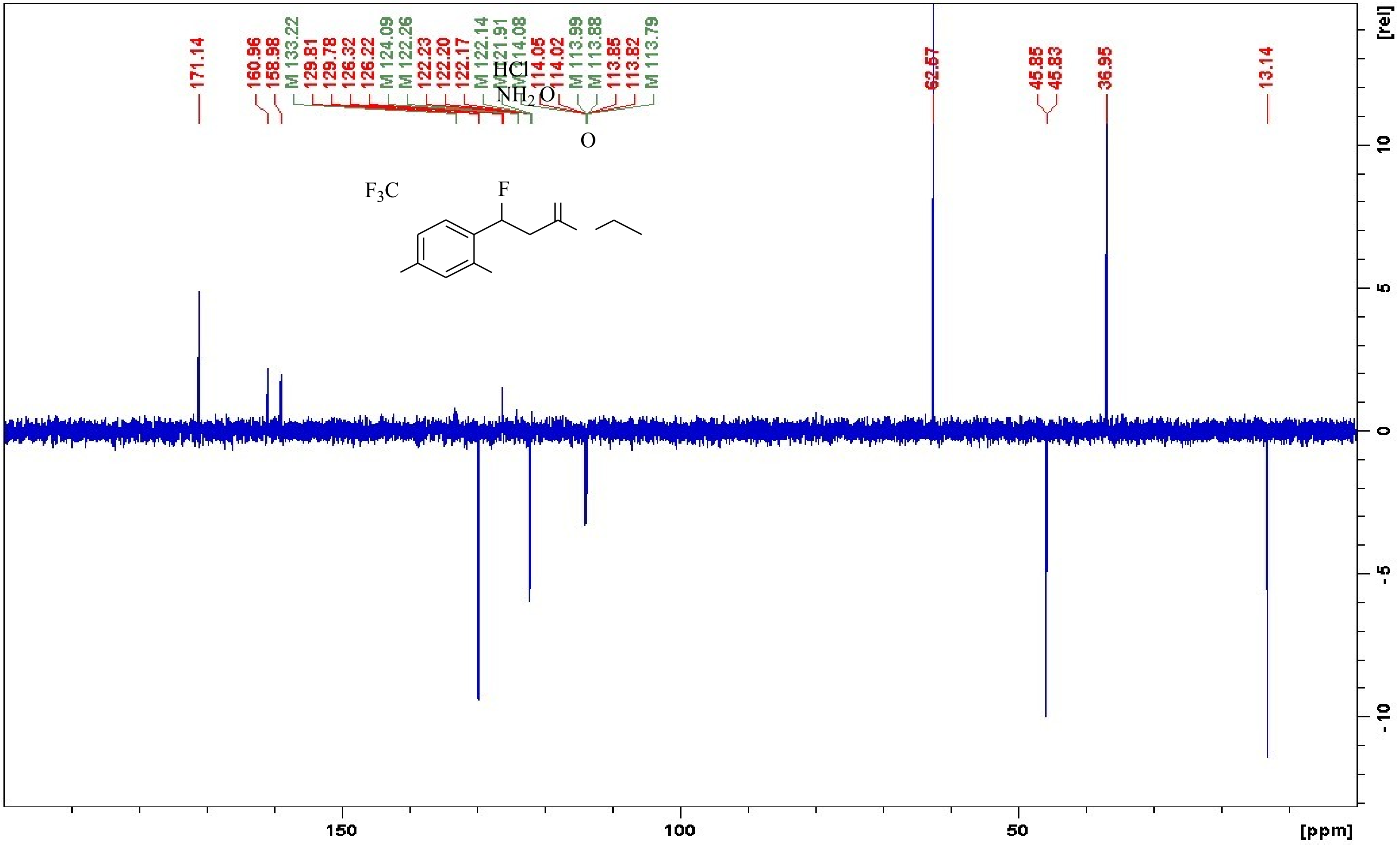

Figure 30. ^{13}C NMR (126 MHz, D_2O) spectra for (±) **3e**

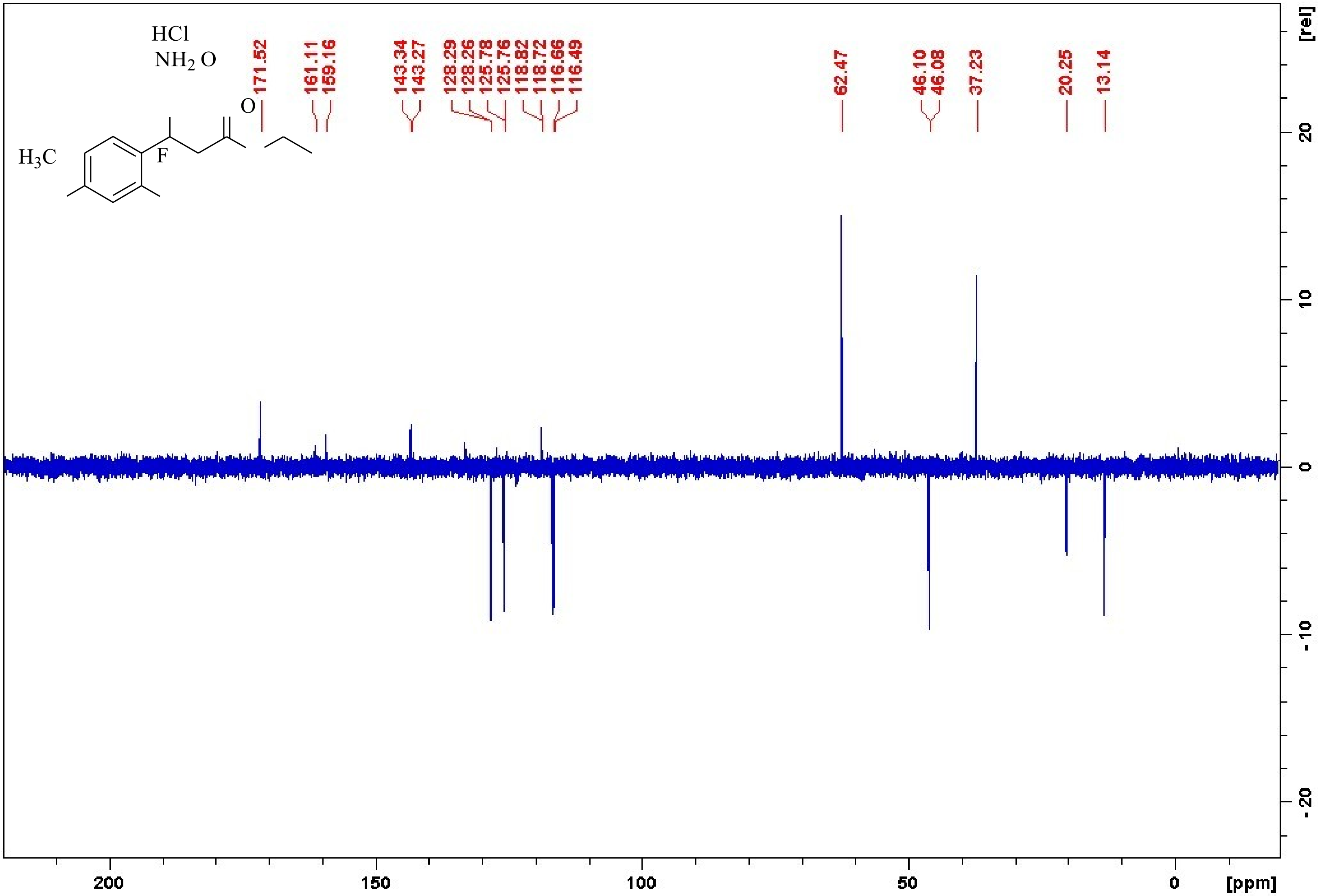

Figure 31. ^{19}F NMR (471 MHz, D_2O) spectra for (±) **3a**

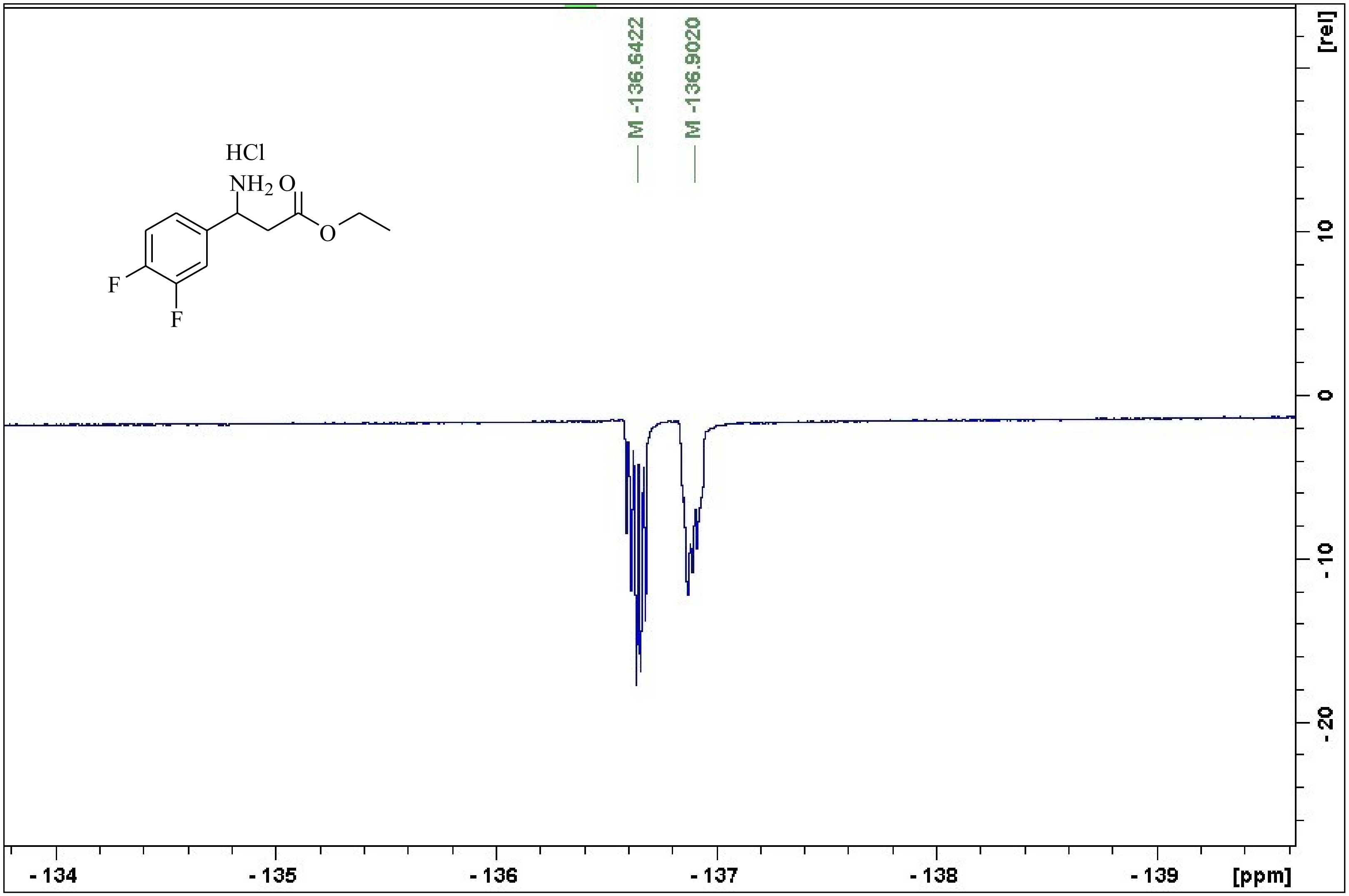

Figure 32. ^{19}F NMR (471 MHz, D_2O) spectra for (±) **3b**

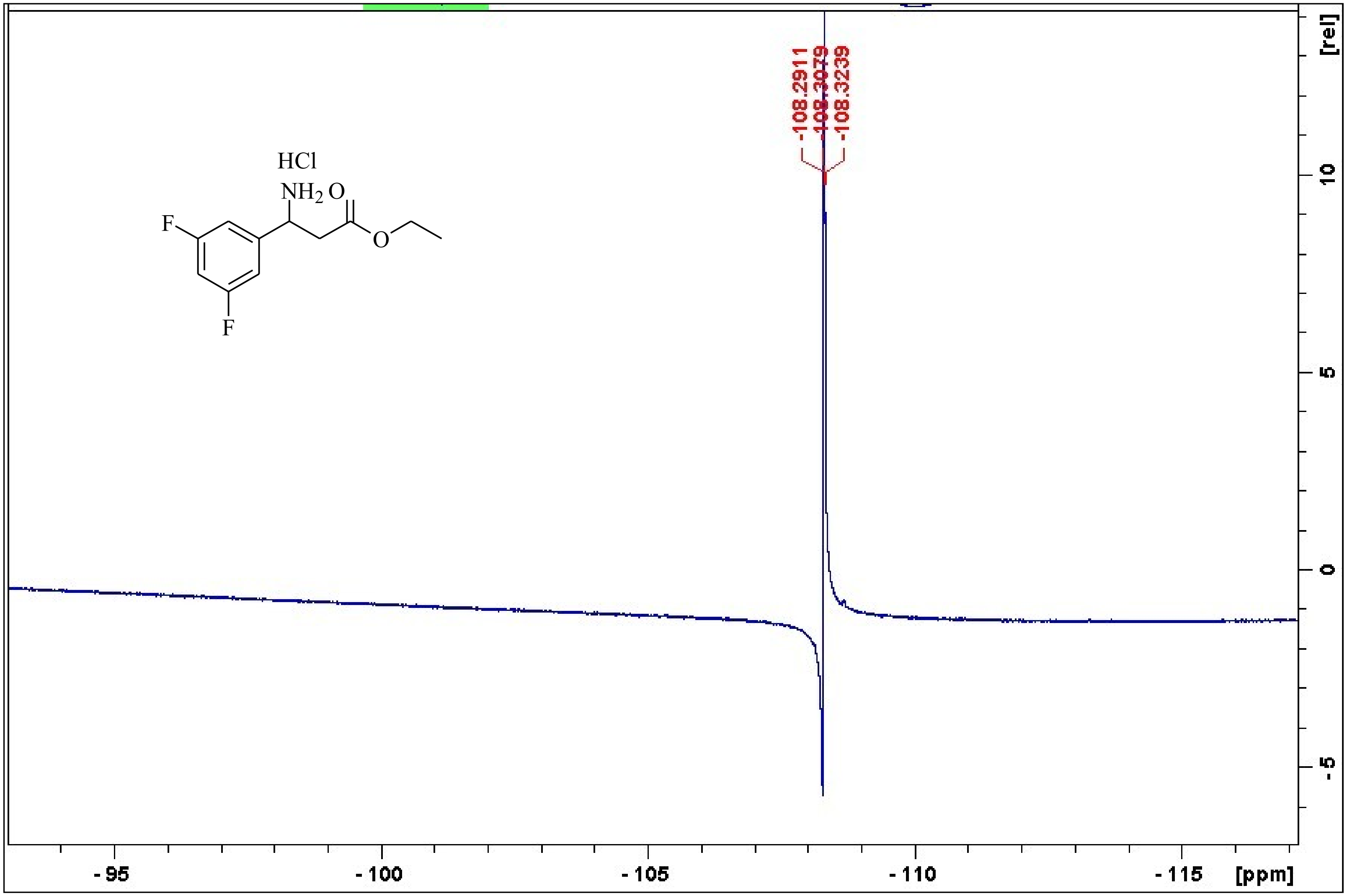

Figure 33. ^{19}F NMR (471 MHZ, D_2O) spectra for (±) **3C**

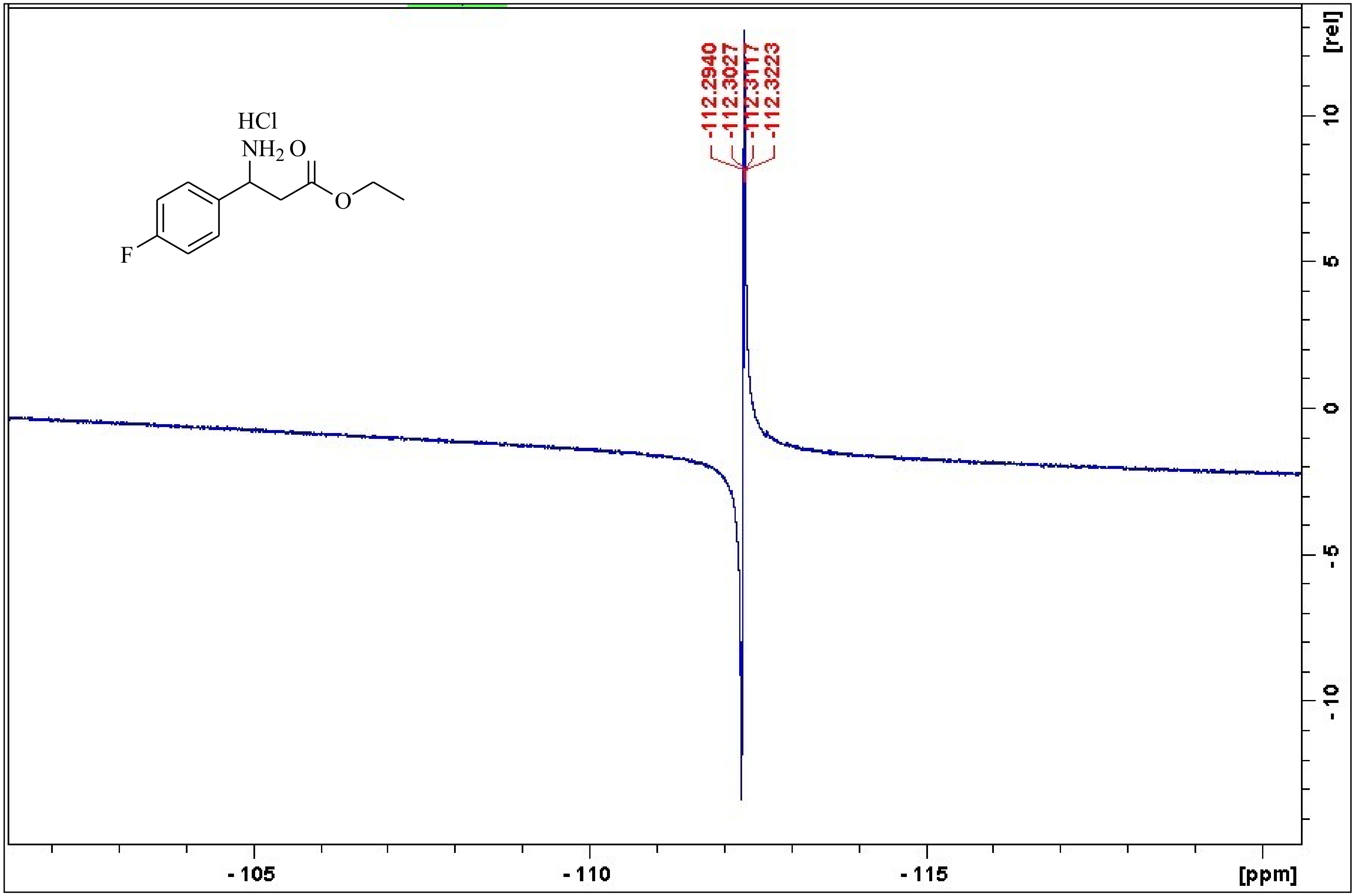

Figure 34. ^{19}F NMR (471 MHz, D_2O) spectra for (±) **3d**

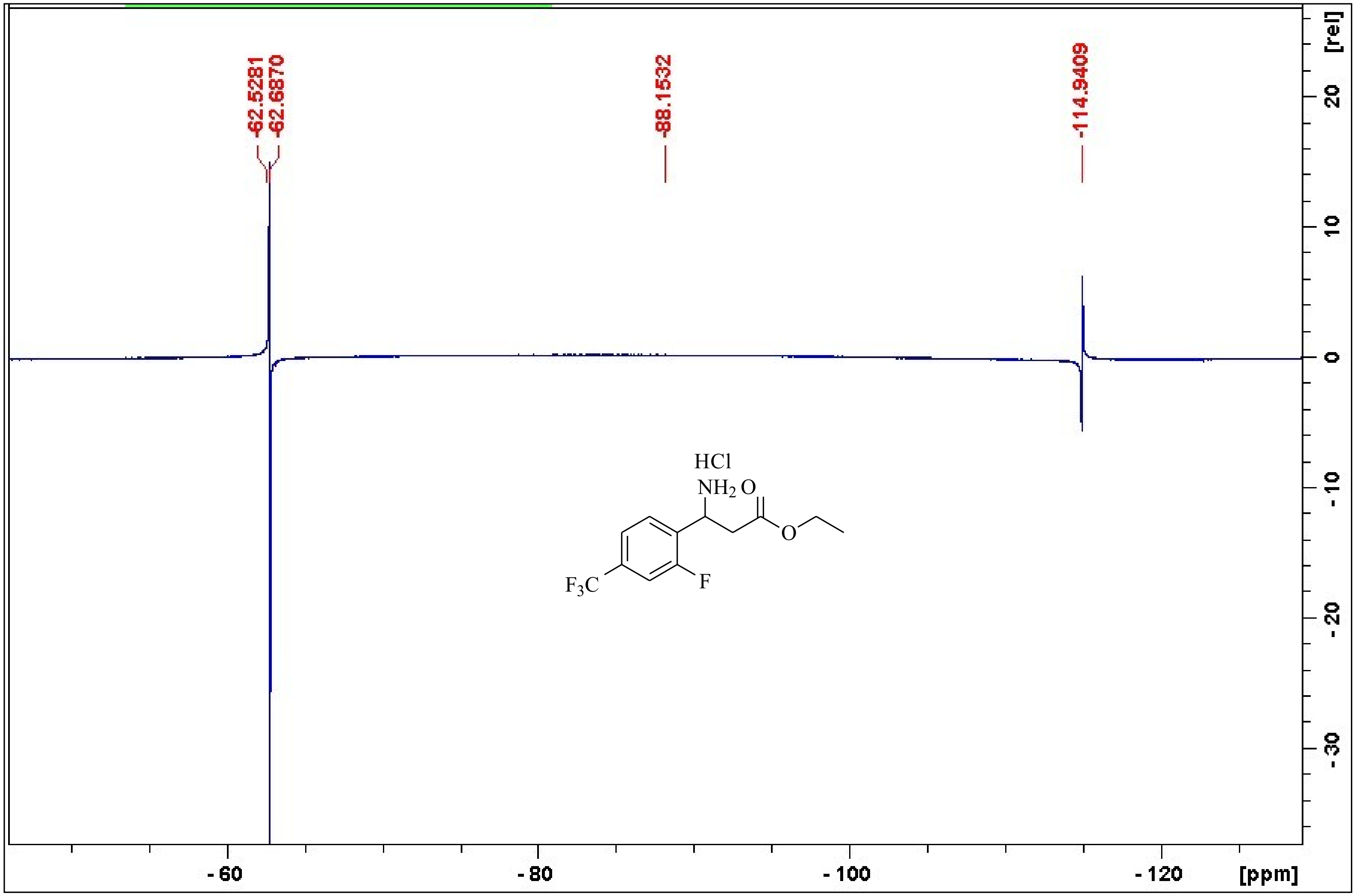

Figure 35. ^{19}F NMR (471 MHz, D_2O) spectra for (±) **3e**

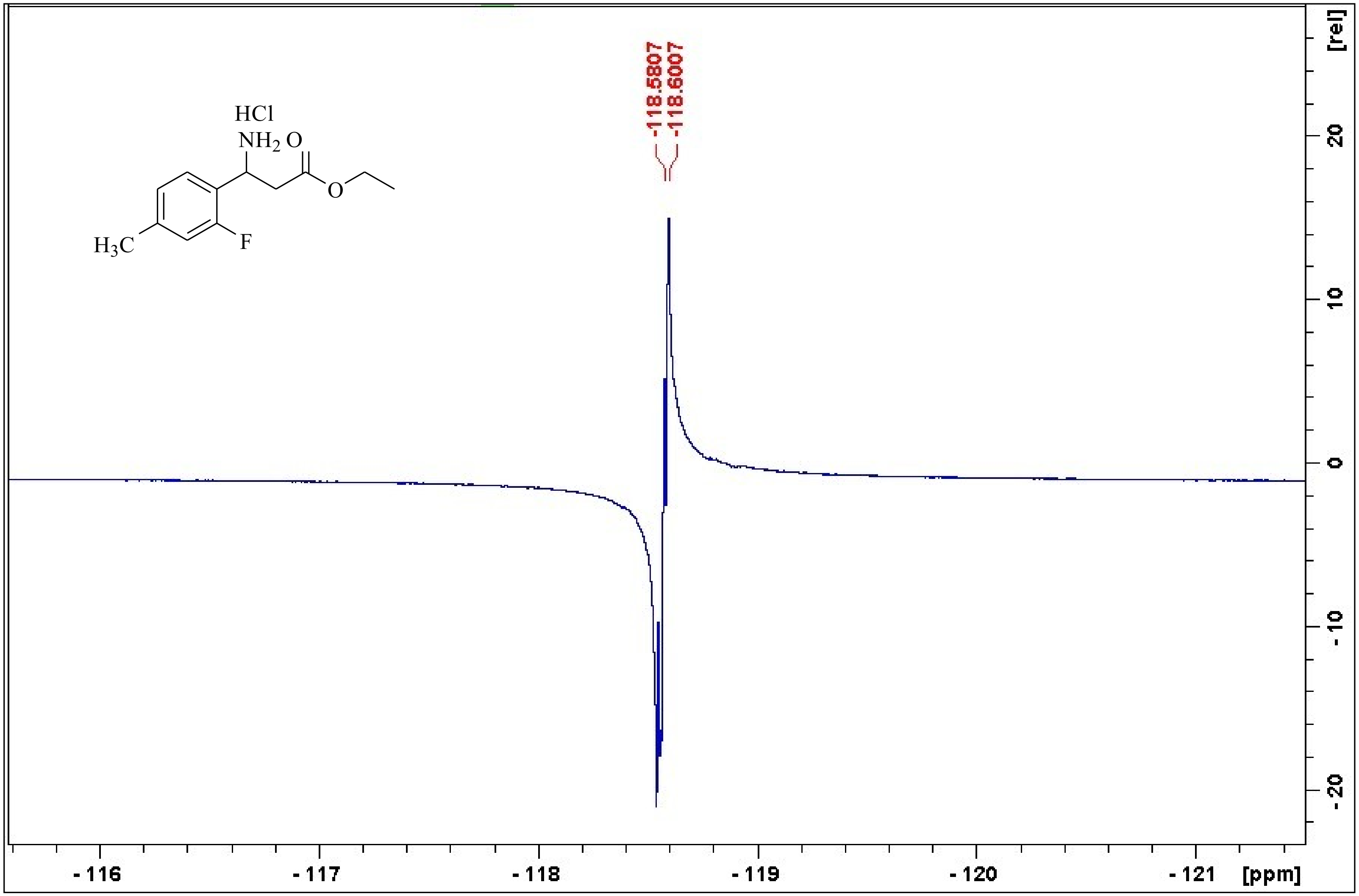

Supplement: Supplementary file 1 [file molecules-25-05990-s001.pdf]
